# Spatially explicit model of the *Cryptosporidium* and *Giardia* disease burden from surface and ground waters in urban and rural areas of the Three Gorges Reservoir watershed in Chongqing, China

**DOI:** 10.1007/s11356-022-24690-2

**Published:** 2022-12-26

**Authors:** Qian Huang, Shan Huang, Bo Li, Yanhong Xiong, Weijie Kuang, Shunxin Xiao, Jianghui Yi, Feng Zhao, Guosheng Xiao

**Affiliations:** 1 College of Biology and Food Engineering, Chongqing Three Gorges University, Wanzhou, 404130 China; 2 College of Grassland Science and Technology, Sichuan Agricultural University, Chengdu, 611130 China

**Keywords:** *Cryptosporidium*, *Giardia*, Three Gorges Reservoir, GloWPa-TGR-Crypt-Giar model, Water quality, Quantitative microbial risk assessment

## Abstract

*Cryptosporidium* and *Giardia* (major causes of diarrhea) are widely distributed in Chinese source waters and threaten human health. A new spatially explicit GloWPa-TGR-Crypt-Giar C1 model is presented to simultaneously estimate mean monthly (oo)cyst concentrations in surface and ground waters in the Three Gorges Reservoir (TGR) watershed. A quantitative risk assessment of protozoal infections considered different source waters, transmission pathways, regions, susceptible subpopulations, and drinking water treatments. Monthly mean *Cryptosporidium* oocyst and *Giardia* cyst concentrations ranged between 0.5–19.3 oocysts/10 L and 0.2–5.0 cysts/10 L in surface water, respectively, and 0.007–0.3 oocysts/10 L and 0.002–0. 2 cysts/10 L in groundwater. The cumulative disease burdens attributable to cryptosporidiosis and giardiasis were, respectively, 5.77×10^−5^ DALYs (disability-adjusted life years/person/year) and 4.63×10^−6^ DALYs in urban areas, and 6.35×10^−4^ DALYs and 8.84×10^−5^ DALYs in rural areas, which were much higher than the reference risk level recommended by the World Health Organization (${10}^{-6}$ DALYs). The annual burden associated with consuming surface water was calculated to be 3.84×10^−4^ DALYs for *Cryptosporidium* and $5.10\times {10}^{-5}$ DALYs for *Giardia*, whereas consuming groundwater entailed the lower burdens (1.26×10^−5^ and 3.50×10^−6^ DALYs, respectively). Most DALYs were a consequence of consumption of directly supplied surface water. Fifty percent of the health burden was carried by immunodeficiency with HIV. Children (0–4 years) were more likely to have an individual disease burden than adults (15–64 years). Males were more susceptible than females. Improving sanitation through adequate ozone and microfiltration treatment should be considered when attempting to reduce disease burden. Sensitivity analysis highlighted the importance of reducing (oo)cyst loads to protect the watershed. The methodology and results described will help in evaluating and reducing the burden of protozoal infection associated with surface and ground waters in the TGR and similar watersheds.

**Graphical Abstract:**

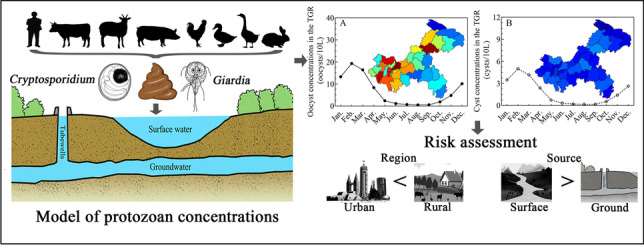

**Supplementary Information:**

The online version contains supplementary material available at 10.1007/s11356-022-24690-2.

## Introduction

*Cryptosporidium* and *Giardia* are critical parasites in the etiology of diarrhea worldwide and often cause waterborne outbreaks. Around the world, the largest disease burden occurs in immunodeficient individuals, particularly children younger than 5 years and HIV/AIDS patients (Liu et al. 2014; Ghafari et al. 2018; Han et al. 2020). The highly contagious oocysts and cysts shed by an infected person or animal are transmitted via the fecal–oral route, by direct or indirect contact (Castro-Hermida et al. 2009; Dixon 2016; Saaed and Ongerth 2019). Contaminated water is a major indirect transmission route since *Cryptosporidium* oocysts and *Giardia* cysts are suited to waterborne transmission. In China, these parasites have also been detected in wastewater (Xiao et al. 2018a), reclaimed water (Zhang et al. 2015), recreational waters (Xiao et al. 2018b), and surface water (Xiao et al. 2012, 2013). However, the occurrence and risk from these protozoa in groundwater in China remain unclear, making it difficult to effectively manage groundwater and develop reasonable standards of water quality.

In contrast to surface water, which is susceptible to fecal contamination from point and nonpoint sources (Huang et al. 2020), groundwater is generally considered to be of higher quality due to the contaminant attenuation capacity of the overlying (sub)soil (Chique et al. 2020). However, field studies and outbreak investigations both indicate that groundwater systems can be significant sources of enteric pathogens including *Cryptosporidium* and *Giardia* (Daniels et al. 2018; Chique et al. 2020). In rural areas of Chongqing (China), approximately 39% of people rely on untreated groundwater (e.g., tubewells) for drinking water (Wang and Hou 2011; Jiang et al. 2013), which raises concerns about exposure to waterborne pathogens arising from fecal contamination. Given the significant knowledge gap about groundwater in China, a sound understanding of pathogen concentrations in surface and ground waters remains essential to guiding future environmental and public health research and policy (Bradford and Harvey 2017). However, determining *Cryptosporidium* and *Giardia* (and other pathogen) concentrations is expensive and time-consuming, and accurate measurement of ambient concentrations of (oo)cysts is difficult, resulting in a scarcity of observational data, especially from developing countries (Efstratiou et al. 2017). Consequently, modeling is a common approach to increasing insight in this area; for example, by pinpointing high concentration hotspots and elucidating the risks and disease burdens associated with waterborne pathogens (Vermeulen et al. 2019; Limaheluw et al. 2019).

Quantitative microbial risk assessment (QMRA) is a useful method of predicting the human health risk from pathogens. It has been widely used to assess the infection risk of *Cryptosporidium* and/or *Giardia* in various types of water (Zhang et al. 2015; Daniels et al. 2018; Han et al. 2020; Xiao et al. 2013, 2018a, b). Co-infections are significantly more fatal to humans than single infections. The death rate due to co-infection in some developing countries is estimated to be one-fifth to one-third (Rodríguez-Morales et al. 2016) and is particularly high for those with HIV (Liu et al. 2014). By the end of 2016, over 400,000 Chinese people were infected with HIV (Feng and Xiao 2011; NHFP 2015; CDC 2016). Han et al. (2020) addressed co-infection with *Cryptosporidium*/*Giardia* and HIV by embedding this into the dose–response relationship for estimating infections and applying age sensitivity coefficients to accurately evaluate the effects of co-infection.

People living in rural areas of China have a significantly higher prevalence of *Cryptosporidium* exposure (1.8–12.9%) than those in urban areas (0–3.7%) because most rural areas are underdeveloped and economically weak (Liu et al. 2020). It is estimated that only 29% of drinking water receives conventional treatment in rural areas of Chongqing (Jiang et al. 2013), compared with nearly 95% in urban areas. Previous studies assumed the treatment of drinking water to be the same in all regions (Xiao et al. 2013; Han et al. 2020), ignoring the different daily (oo)cyst intakes of the urban and rural populations and different transmission pathways (direct consumption or piped supply of treated water), leading to underestimation of cryptosporidiosis and giardiasis in rural areas.

This study focuses on the Three Gorges Reservoir (TGR), which is located on the upper reaches of the Yangtze River. This is one of the world’s largest comprehensive hydropower projects and a major source of drinking water for the region which includes a highly urbanized coastal area (He et al. 2011). Human and livestock feces enter the TGR directly with minimal or no pre-treatment, leading to serious *Cryptosporidium* and *Giardia* pollution (Xiao et al. 2013; Liu et al. 2019; Huang et al. 2020). However, there is currently no comprehensive understanding of the risk and disease burden attributable to parasitic pathogens consumed via TGR surface and ground waters. It is necessary to estimate (oo)cyst concentrations to conduct any in-depth analysis of the health risks posed by these pathogens. In contrast to previous risk assessments, this study presents the following outputs: (1) a new spatially explicit GloWPa-TGR-Crypt-Giar C1 model to simultaneously estimate mean monthly (oo)cyst concentrations in surface and ground waters in the TGR watershed, (2) analysis of source water, transmission pathway, and regional impacts on diarrheal disease burdens, and (3) analysis of health impacts associated with susceptible subpopulations and drinking water treatment processes. This methodology and data will help in the evaluation and reduction of the burden of protozoal infection associated with surface and ground waters in the TGR and similar watersheds.

## Materials and methods

### Study area and population

The TGR occupies the lower section of the upper reaches of the Yangtze River (28° 30′ to 31° 44′ N and 105° 44′ to 111° 39′ E) and is the world’s largest hydropower project and a major source of drinking water to an area that includes a highly urbanized coast. The Chongqing population served by the TGR includes approximately 29.72 million residents (58.3% being urbanized) with an age composition of 5.7% (≤ 4 years), 5.3% (5–9 years), 5.4% (10–14 years), 71.7% (15–64 years), and 11.9% (≥ 65 years). More details are given in Table S1.

### Calculation of protozoa concentrations

**Fig. 1:**
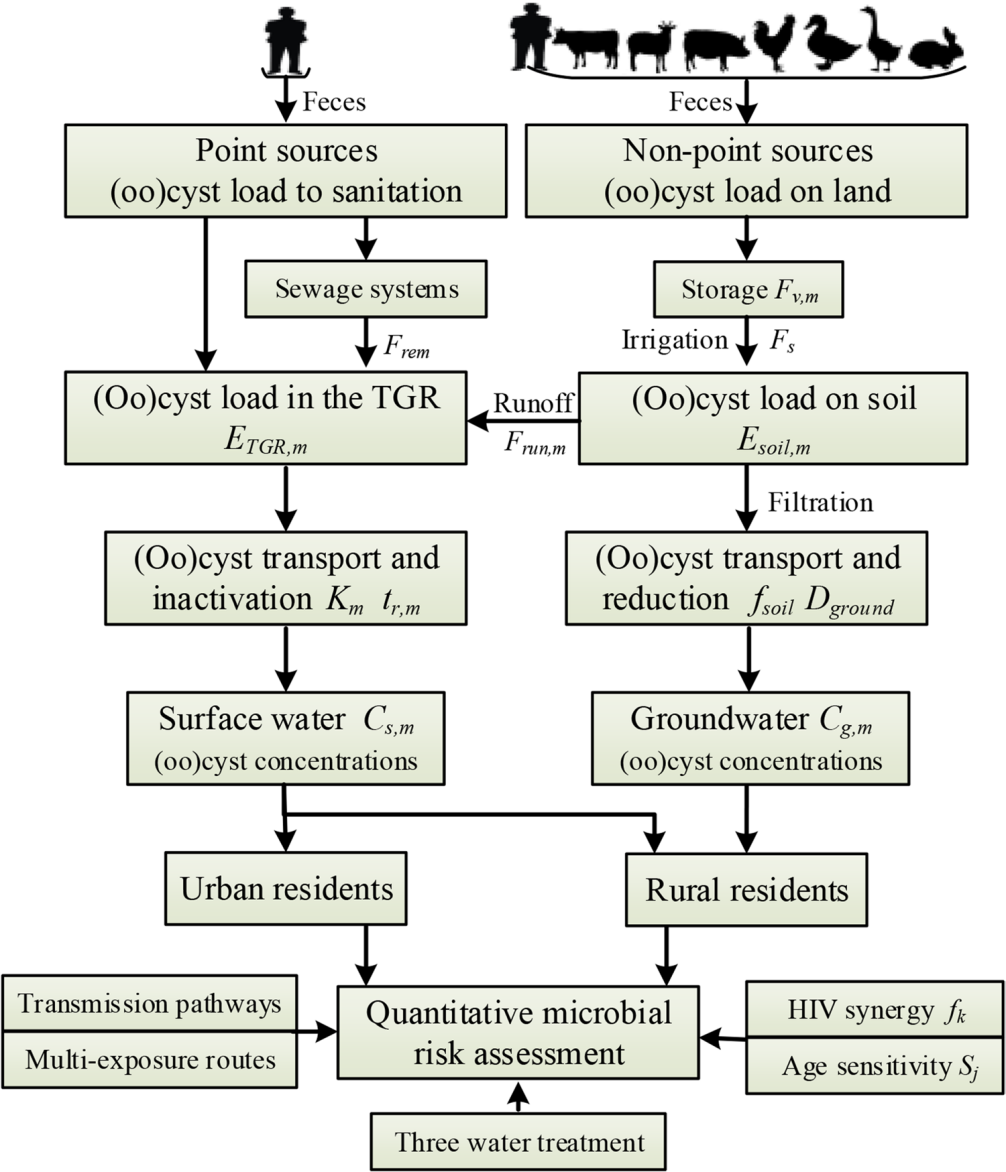
Schematic of the components of the GloWPa-TGR-Crypt-Giar C1 model illustrating the flow of (oo)cysts from point and nonpoint sources to surface water and groundwater in the TGR watershed and the health risks of *Cryptosporidium* and *Giardia* in urban and rural areas

This study constructed a modified GloWPa-TGR-Crypt-Giar C1 model based on the original GloWPa-TGR-Crypto model described in detail by Hofstra et al. (2013) and Huang et al. (2020) and focused on model modifications and input variable changes. Figure 1 shows a schematic of the model components. Briefly, the GloWPa-TGR-Crypt-Giar C1 model simulates loads from point sources (comprised of urban and some rural residents) and nonpoint sources (comprised of livestock and some rural residents) of *Cryptosporidium* and *Giardi*a to calculate surface and groundwater (oo)cyst concentrations. This study modifies the original model in several ways. Firstly, the annual (oo)cyst loads are divided by twelve as an estimate of monthly loads. Input parameters are separated into 12 months to enable operation of the model at monthly intervals. Secondly, where data are available, a statistical distribution is applied to the input parameters based on an extensive literature review (more details of the distribution can be found in the supplemental files). This substitutes for a fixed value and captures some of the inherent uncertainty and variability of the data. Thirdly, the modified model simultaneously estimates mean monthly (oo)cyst concentrations in surface and ground waters with associated 95% confidence intervals (95% CI) in the TGR watershed in Chongqing. The study area was subdivided at urban and rural district/county level, and Monte Carlo simulation produced 10,000 estimated values. For concentration used in the QMRA, only the zoonotic *Cryptosporidium parvum*, *Cryptosporidium hominis*, and *Giardia lamblia* assemblages A and B were considered, as these are the most common waterborne causes of infection in humans (Sprong et al. 2009; Limaheluw et al. 2019).

#### Sources of Cryptosporidium and Giardia loads

The GloWPa-TGR-Crypt-Giar C1 model simulated monthly human and livestock *Cryptosporidium* and *Giardia* loads from point and nonpoint sources into the TGR and soils (Hofstra et al. 2013; Huang et al. 2020). The model computed monthly spatial outputs from human or livestock populations, fecal pathogen shedding, sanitation coverage, wastewater treatment removal, storage survival, and surface runoff. Unlike in Huang et al. (2020) where a constant annual (oo)cyst excretion per person in developing countries was used, we calculated annual (oo)cyst excretion per person by multiplying annual feces per person in China, daily (oo)cyst excretion rate per infected person, and cryptosporidiosis and giardiasis prevalence in China. A statistical distribution for monthly storage survival and surface runoff parameters were applied to distinguish the effects of seasonal meteorological parameters on the loads. Thus, differentiated sanitation management events and pathways were integrated into this new GloWPa-TGR-Crypt-Giar C1 model (Fig. 1). More details are provided in Supporting Information Section S1 and Table S2.

#### Protozoa concentrations in the TGR

(Oo)cyst loads from point and nonpoint sources into the TGR, survival fraction of (oo)cysts during transport in water, and river discharge data were used to build the GloWPa-TGR-Crypt-Giar C1 model for simulating concentrations in surface water (Fig. 1, Table 1, and Table S2). Like Vermeulen et al. (2019), (oo)cysts survival in rivers for a particular month was estimated from the literature and the total inactivation rate (*K*_*m*_) developed by summing rate order die-offs due to temperature, solar radiation, and sedimentation, before the loads were routed downstream. River depth (m), width (m), and flow velocity (${\text{m}}^{3}{{\text{s}}}^{-1}$) calculated from monitoring and the literature enabled estimation of water residence time (${\text{t}}_{\mathrm{r,m}}$) (Vermeulen et al. 2019; Okaali et al. 2021). As the TGR is a channel reservoir (on the largest river in China—the Yangtze), monthly (oo)cyst survival concentrations were estimated using the approach of Vermeulen et al. (2019). The total inactivation rate and water residence time were calculated using Eqs. 6 and 7 in Table S2. Compared to previous studies, input parameters such as water temperature, surface solar radiation, and water level were utilized to create a monthly probability distribution function instead of an annual fixed value to calculate protozoa concentrations more accurately over time. Total monthly mean concentrations of (oo)cysts (${\text{C}}_{\mathrm{s, m}}$) were calculated from discharge (${\text{Q}}_{\mathrm{m}}$) using Eq. 1 (Table 1).

**Table 1:** Formulas and parameters for calculating (oo)cyst concentrations and assessing risk

No. Eq	Model description	Calculation formula	Parameter description	Parameter values
1	Concentrations in the TGR	${C}_{s,m}=\frac{{E}_{TGR,m}\times {e}^{-{K}_{m}\times {t}_{r,m}}}{{Q}_{m}}$	${{C}}_{\mathrm{s,m}}$: concentration of (oo)cysts in month *m* in the TGR ((oo)cysts ${\text{/m}}^{3}$); ${{E}}_{{\mathrm{TGR}}\text{, m}}$: (oo)cyst loads to the TGR in month *m* ((oo)cysts /month) ${{K}}_{\mathrm{m}}:$ total inactivation rate in month *m* (${\text{day}}^{-1}$); ${{t}}_{\mathrm{r, m}}$: residence time of (oo)cyst in Chongqing section of the TGR in month *m* (days); ${{Q}}_{\mathrm{m}}$: average reservoir inflow discharge in month *m* (${\text{m}}^{3}\text{/month}$)	${{E}}_{{\mathrm{TGR}}\mathrm{, m}}$: was in Table S2; ${{K}}_{\mathrm{m}}$ and ${{t}}_{\mathrm{r, m}}$: was in Table S2; ${{Q}}_{\mathrm{m}}$: was in Table S3
2A 2B 2C	Concentrations in groundwater	${F}_{\mathrm{filt}}={10}^{-{f}_{\mathrm{soil}}}\times {D}_{\mathrm{ground}}$ ${E}_{\mathrm{ground},m}={E}_{\mathrm{soil},\mathrm{m}}\times {F}_{\mathrm{filt}}$ ${C}_{g,m}=\frac{{E}_{\mathrm{ground},m}}{{S}_{\mathrm{area}}\times {P}_{m}}$	${{C}}_{\mathrm{g, m}}$: concentration of (oo)cysts in month *m* in groundwater ((oo)cysts ${\text{/m}}^{3}$); ${{F}}_{\mathrm{filt}}$: the loss rate coefficient during filtration; ${\text{f}}_{\mathrm{soil}}$: reduction factor for transport in soil; ${{D}}_{\mathrm{ground}}$: depth of groundwater used for drinking in China (m); ${{E}}_{{\mathrm{ground}}\text{, m}}$: (oo)cyst loads in the groundwater in month *m* ((oo)cysts /month); ${{E}}_{{\mathrm{soil}}\text{, m}}$: (oo)cyst loads on soil in month *m* ((oo)cysts /month); ${{S}}_{\mathrm{area}}$: sown area in Chongqing (${\text{m}}^{2}$); ${{P}}_{\text{m}}$: precipitation in month *m* (mm)	${{f}}_{\mathrm{soil}}$: was in Table S4; ${{D}}_{\mathrm{ground}}$: was in Table S4; ${{E}}_{{\mathrm{soil}}\text{, m}}$: was in Table S2; ${{S}}_{\mathrm{area}}$: was in Table S4 ${{P}}_{\mathrm{m}}$: was in Table S3
3	Daily (oo)cyst intake	$D={C}_{m}\times {10}^{-\left({R}_{c}+{R}_{o}+{R}_{mf}\right)}\times FI\times {V}_{i}$	${{D}}$: daily ingest (oo)cysts ($\text{(}{\mathrm{oo}}\text{)cysts/day}$); ${{R}}_{\mathrm{c}}$: log removal by conventional treatment; ${{R}}_{\mathrm{o}}$: log removal by ozone; ${{R}}_{\text{mf}}$: log removal by MF; *FI*: fraction of viable (oo)cysts able to cause host infection (%); ${{V}}_{\mathrm{i}}$: volume of water ingested from exposure route *i* ($\text{L/day}$)	Values summarized in Table S4
4	Infection risk per day(time)	${P}_{\mathrm{age},\text{inf}}=\left(1-{e}^{-r\times D}\right)\times {S}_{j}\times {f}_{k}$	${{P}}_{{\text{age}}\mathrm{,}{\text{inf}}}$: daily infection rate modified by age sensitivity; ${{r}}$: dose–response parameter; ${{S}}_{\mathrm{j}}$: sensitivity parameters to (oo)cysts in different age groups *j*; ${{f}}_{\text{k}}$: infection-enhancing coefficient of independent or synergistic effect of HIV	Values summarized in Tables S1 and S4
5A 5B	Risk characterization	${P}_{\mathrm{ill},\text{year}}=\left[1-{\left(1-{P}_{\mathrm{age},\text{inf}}\right)}^{{T}_{\mathrm{year}}}\right]\times {P}_{\mathrm{ill},\text{inf}}$ ${P}_{\mathrm{cfr},\text{year}}={P}_{\mathrm{ill},\text{year}}\times {P}_{\mathrm{cfr}}$	${{P}}_{\text{ill,year}}$: annual probability of illness; ${{T}}_{\text{year}}$: frequency of exposures per year; ${{P}}_{\text{ill,inf}}$_:_ probability of developing illness given infection; ${{P}}_{\text{cfr,year}}$: annual probability of mortality; ${{P}}_{\text{cfr}}$: probability of developing mortality given illness	Values summarized in Table S4
6	Disease burden	$\mathrm{YLLs}=\sum_{i=1}^{3}\sum_{j=1}^{n}\sum_{k=1}^{2}{N}_{j,k}\times {F}_{i}\times {P}_{{\mathrm{cfr},\text{year}}_{j,k}}\times {e}_{j}$ $\mathrm{YLDs}=\sum_{i=1}^{3}\sum_{j=1}^{n}\sum_{k=1}^{2}\sum_{l=1}^{3}{N}_{j,k}\times {F}_{i}\times {P}_{{\mathrm{ill},\text{year}}_{j,k}}\times {P}_{{\mathrm{sym}}_{l}}\times {L}_{l}\times {W}_{l}$	${{N}}_{\text{j,k}}$: population at age *j* in group *k* (immunocompetence and immunodeficiency); ${{F}}_{\text{i}}$_:_ fraction of exposure population in exposure routes *i*; ${{e}}_{\text{j}}$: standard life expectancy at age *j* (day); ${{P}}_{{\text{sym}}_{\text{l}}}$: proportions of acute cryptosporidiosis and giardiasis at symptom *l*; ${{L}}_{\text{l}}$: duration of acute cryptosporidiosis and giardiasis at symptom *l* (day); ${{W}}_{\text{l}}$: disability weights of acute gastroenteritis at symptom *l*	Values summarized in Tables S1 and S4

#### Protozoa concentrations in groundwater

For nonpoint sources of (oo)cysts loaded onto agricultural land via fertilization when raining, a portion was assumed to be transported to surface water via surface runoff (Eq. 4C in Table S2) and a portion transported to groundwater via subsurface filtering through the soil (Fig. 1). Literature on transport of *Cryptosporidium* in soils was reviewed. Precipitation events of sufficient intensity erode the feces and release oocysts onto the wetted soil surface which then infiltrate the subsurface (Tate et al. 2000; Atwill et al. 2002; Santamaría et al. 2011). *Cryptosporidium* and *Giardia* transported into the subsurface, survival fraction in the groundwater, sown area, and precipitation data were used to build the first GloWPa-TGR-Crypt-Giar C1 model for simulating groundwater concentrations at district/county level. Atwill et al. (2002) and Santamaría et al. (2011) showed that transport of oocysts through the soil matrix largely depends on the soil reduction factor (${{f}}_{\text{soil}}$) representing a reduction of 1 to 2 log units of oocysts per meter of travel. The total loss rate coefficient (${{F}}_{ \, {\text{filt}}}$) during transport in soils followed Atwill et al. (2002) and Santamaría et al. (2011) by multiplying the soil reduction factor (${{f}}_{\text{soil}}$) and the depth of groundwater (${{D}}_{\text{ground}}$) (Eq. 2A, Table 1). Total loading in the groundwater (${{E}}_{\text{ground}}$) was calculated and adjusted for groundwater survival using the loss rate coefficient (Eq. 2B, Table 1). Total monthly concentrations in the groundwater were calculated from sown area (${{S}}_{\text{area}}$) and precipitation (${{P}}_{\text{m}}$) (Eq. 2C, Table 1).

### Quantitative microbial risk assessment

#### Exposure assessment

Source water (oo)cyst concentrations were estimated using the GloWPa-TGR-Crypt-Giar C1 model. Unlike previous models (Xiao et al. 2012, 2013; Han et al. 2020), this study took account of surface and ground water, estimating risk to the urban and rural populations from different water sources, treatments, and exposure routes. Of the urban population, 94.8% had access to a piped supply of treated surface water (http://tjj.cq.gov.cn//tjnj/2014/indexch.htm). Of the rural population, 12.9% and 16.1% consumed a piped supply of treated surface or ground water, respectively, while 31.7% and 39.3% directly consumed untreated surface or ground water, respectively (Wang and Hou 2011; Jiang et al. 2013). It was assumed that each individual consumed water from a single source. Conventional drinking water treatment in China consists of coagulation, sedimentation, filtration, and additional disinfection using chlorine (Xiao et al. 2012). All piped water supplies were assumed to have received conventional treatment (Xiao et al. 2012). Furthermore, minimization of microbial risk was explored through QMRAs of three piped water treatment scenarios: (1) conventional treatment, (2) advanced treatment using ozone, and (3) advanced treatment using ozone and microfiltration.

Exposure and ingestion volumes were categorized into three routes: incidental ingestion from tooth brushing, food, and dishwashing (0.039 L/day) (An et al. 2011), direct drinking (0.53–1.50 L/day) (Wang and Duan 2016; Zhao and Duan 2013, 2016), and water swallowed during swimming (0.38–0.82 mL/min) (Dufour et al. 2006) as shown in Eq. 3 (Table 1). Detailed descriptions and age-related values are given in Table S4. The frequency of incidental intake and drinking intake were estimated to be 365 days/year for all populations, and swimming to be 96 times/year for swimming populations (Table S5) (Han et al. 2020). The viability of all (oo)cysts was modeled as a beta distribution with estimated mean viability of 90% for *Cryptosporidium* and 100% for *Giardia* (Table S4) (Xiao et al. 2018b).

#### Dose–response assessment

Probability of infection was estimated using exponential dose–response models with infectivity constants (*r*) of 0.09 for *Cryptosporidium* (USEPA 2006) and 0.059 for *Giardia* (Han et al. 2020). To account for the variability, a lognormal distribution was used to describe the infectivity constant of each parasite (Brouwer et al. 2017). Age sensitivity to infection was represented by the constant ${{S}}_{\text{j}}$, which is an infectivity adjustment factor for children and the elderly relative to adults (Han et al. 2020). Infection mode was separated into single infections (*Cryptosporidium* or *Giardia* alone) and co-infections (*Cryptosporidium* or *Giardia* alongside HIV) and was represented by the constant ${{f}}_{\text{k}}$. The probability of infection per exposure event was calculated for *Cryptosporidium* and *Giardia* using Eq. 4 (Table 1).

#### Risk characterization

The annual risk of illness was calculated from the risk of infection and the annual probability of illness (Eq. 5A, Table 1). The annual risk of mortality was calculated by multiplying the risk of illness by the probability of mortality (Eq. 5B, Table 1).

#### Disease burden

Gastroenteritis caused by *Cryptosporidium* or *Giardia* may result in a reduction in survival time (premature death) and quality of life, or both. Severity is classified as: (1) mild, i.e., no need to visit a general practitioner, (2) outpatient, i.e., needs to visit a general practitioner, (3) hospitalized, and (4) severe symptoms, i.e., fatal (Xiao et al. 2018b). The annual disease burden (Eq. 6, Table 1) is represented by disability-adjusted life years (DALY), which consists of years of life lost (YLL) and years lived with disability (YLD). The cumulative health risk was determined for two population subgroups: immunocompetence and immunodeficiency (with five age subgroups).

### Model implementation

Probability distribution fitting, determination of protozoa concentrations, Monte Carlo simulations, and sensitivity analyses were conducted using @RISK version 6.3. With each input parameter (summarized in Table S4), 10,000 iterations were randomly sampled for each model to account for uncertainty and variability.

## Results

### (Oo)cyst concentrations in surface and ground waters

**Fig. 2:**
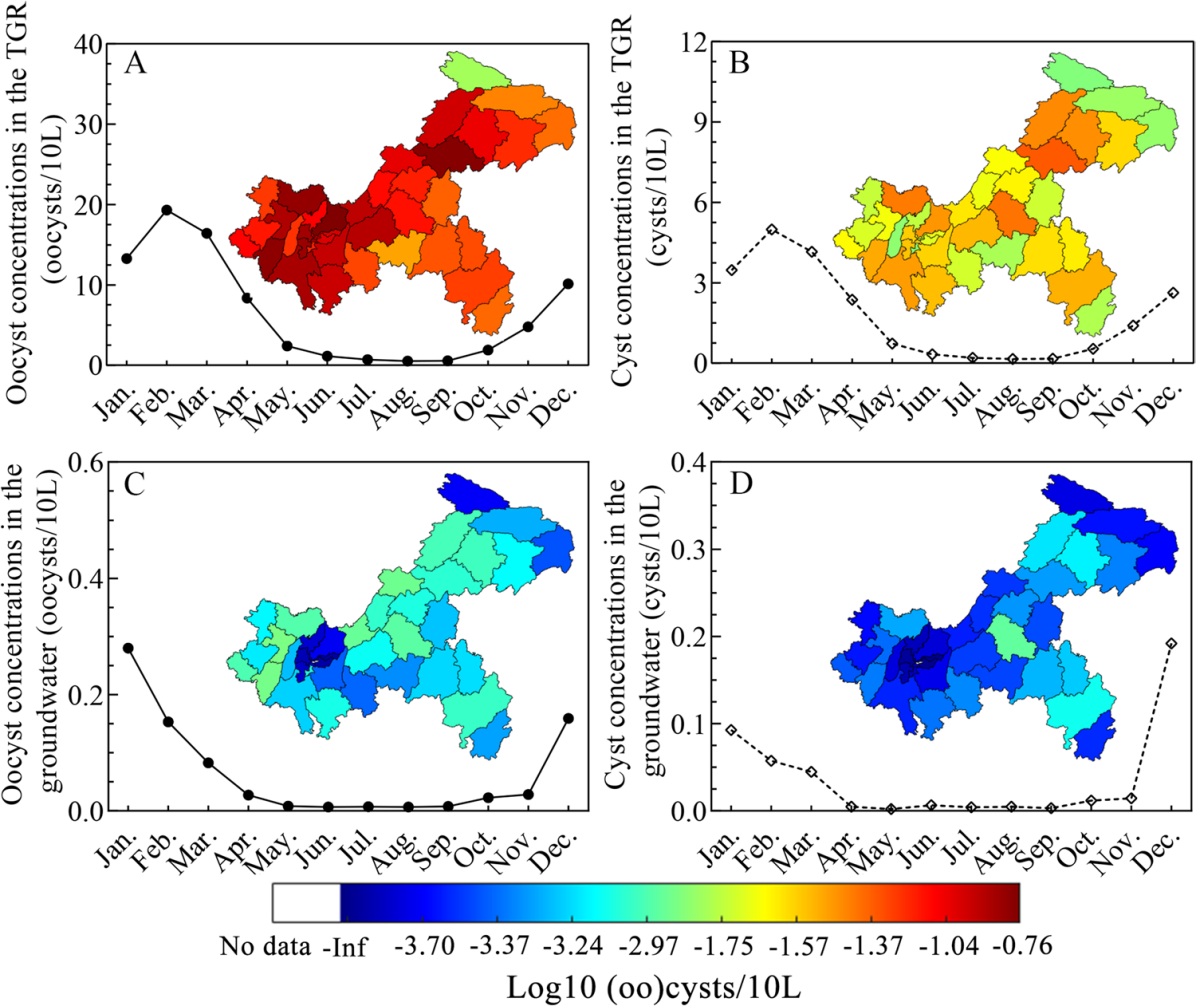
Monthly (solid line) and annual (map) mean concentrations of (oo)cysts in surface water (TGR) and groundwater in Chongqing. **A** *Cryptosporidium* in the TGR, **B** *Giardia* in the TGR, **C** *Cryptosporidium* in groundwater, and **D** *Giardia* in groundwater

Monthly mean concentrations varied from 0.5 (95% CI: 0.06 $-$ 1.6) to 19.3 (95% CI: 6.2 $-$ 42.8) *Cryptosporidium* oocysts/10 L and from 0.2 (95% CI: 0.01 $-$ 0.5) to 5.0 (95% CI: 1.4 $-$ 11.5) *Giardia* cysts/10 L in the TGR surface water (Fig. 2A, B, Table S6). Monthly mean concentrations varied from 0.007 $\left(95\% \mathrm{CI}:0-1.1\times {10}^{-12}\right)$ to 0.3 ($95\% \mathrm{CI}:0-2.64\times {10}^{-11}$) oocysts/10 L and 0.002 $95\% \mathrm{CI}:0-8.4\times {10}^{-13}$ to 0.2 $95\% \mathrm{CI}:0-1.13\times {10}^{-11}$ cysts/10 L in groundwater (Fig. 2C, D, Table S7). The model produced a spatial distribution of (oo)cyst concentrations by district*.* In the TGR, annual mean concentrations ranged from ${10}^{-2}$ to ${10}^{-1}$ oocysts/10 L/district (Fig. 2A) and ${10}^{-3}$ to ${10}^{-2}$ cysts/10 L/district (Fig. 2B). Regions with highest concentrations in the TGR area were the densely populated Wanzhou, Yubei, and Jiulongpo districts. Annual mean concentrations in groundwater ranged from ${0}$ to ${10}^{-3}$ (oo)cysts/10 L/district (Fig. 2C, D). Regions with highest concentrations in the groundwater were in rural areas with large human and livestock populations, such as Tongliang, Yongchuan, and Fengdu districts.

### Morbidity in urban and rural areas

Estimated annual morbidity of diarrhea attributable to *Cryptosporidium* and *Giardia* infections via various routes and groups are listed in Table 2 (piped water with conventional treatment). The risk of morbidity was $6.81\times {10}^{-2}$  $95\% \mathrm{CI}:8.41\times {10}^{-3}$ to $1.99\times {10}^{-1}$ per person per year (pppy) for *Cryptosporidium* and $9.96\times {10}^{-3}$ pppy $95\% \mathrm{CI}:5.28\times {10}^{-4}$ to $3.57\times {10}^{-2}$ for *Giardia*. The highest morbidity was from incidental intake, while swimming posed the lowest morbidity for *Cryptosporidium* and *Giardia*. Annual cases caused by *Cryptosporidium* and *Giardia* were estimated to be $2.02\times {10}^{6}$ $95\% \mathrm{CI}:2.50\times {10}^{5}$ to $5.91\times {10}^{-6}$ and $2.96\times {10}^{5}$ $95\% \mathrm{CI}:1.57\times {10}^{4}$ to $1.06\times {10}^{-6}$, respectively (Table 2). Cases in rural areas were nine-fold higher than in urban areas for *Cryptosporidium* and 13-fold for *Giardia*, which significantly affected the distribution of annual cases. The risk of morbidity for immunodeficiency was $1.95\times {10}^{1}$ pppy $95\% \mathrm{CI}:2.57\times {10}^{-2}$ to $4.48\times {10}^{-1}$ for *Cryptosporidium* and $3.09\times {10}^{-2}$ pppy $95\% \mathrm{CI}:1.98\times {10}^{-3}$ to $1.03\times {10}^{-1}$ for *Giardia*, which are much higher than for immunocompetence: $7.05\times {10}^{-2}$ pppy ($95\% \mathrm{CI}:8.45\times {10}^{-3}$ to $2.13\times {10}^{-1}$) for *Cryptosporidium* and $9.96\times {10}^{-3}$ pppy $\left(95\% \mathrm{CI}:5.26\times {10}^{-4} \mathrm{to} 3.57\times {10}^{-2}\right)$ for *Giardia*. In general, the majority of cases resulted from direct consumption of surface water in rural populations.

**Table 2:** Simulated morbidity caused by *Cryptosporidium* and *Giardia* by exposure routes and subgroups in the TGR watershed, and cases per person per year (pppy) per group

		*Cryptosporidium*		*Giardia*	
Exposure route	Total population in group $\times {10}^{4}$	Number of cases (95% CI^a^)	Cases/pppy in group (95% CI)	Number of cases (95% CI)	Cases/pppy in group (95% CI)
Incidental intake	2971.63	$1.62\times {10}^{6}\left(1.37\times {10}^{5}-5.23\times {10}^{6}\right)$	$5.66\times {10}^{-2}\left(4.69\times {10}^{-3}-1.85\times {10}^{-1}\right)$	$2.01\times {10}^{5}\left(8.28\times {10}^{3}-7.82\times {10}^{5}\right)$	$6.94\times {10}^{-3}\left(2.82\times {10}^{-4}-2.62\times {10}^{-2}\right)$
Direct drinking	2971.63	$3.79\times {10}^{5}\left(1.14\times {10}^{5}-6.38\times {10}^{5}\right)$	$1.31\times {10}^{-2}\left(3.93\times {10}^{-3}-2.23\times {10}^{-2}\right)$	$8.86\times {10}^{4}\left(6.89\times {10}^{3}-2.66\times {10}^{5}\right)$	$3.07\times {10}^{-3}\left(2.36\times {10}^{-4}-9.31\times {10}^{-3}\right)$
Swimming	2971.63	$7.82\times {10}^{4}\left(4.21\times {10}^{3}-2.86\times {10}^{5}\right)$	$2.73\times {10}^{-3}\left(1.45\times {10}^{-4}-1.01\times {10}^{-2}\right)$	$1.04\times {10}^{4}\left(3.62\times {10}^{2}-4.13\times {10}^{4}\right)$	$3.60\times {10}^{-4}\left(1.21\times {10}^{-5}-1.43\times {10}^{-3}\right)$
Group					
Urban	1732.46	$2.03\times {10}^{5}\left(1.08\times {10}^{4}-7.54\times {10}^{5}\right)$	$1.17\times {10}^{-2}\left(6.24\times {10}^{-4}-4.35\times {10}^{-2}\right)$	$2.10\times {10}^{4}\left(5.75\times {10}^{2}-8.31\times {10}^{4}\right)$	$1.21\times {10}^{-3}\left(3.32\times {10}^{-5}-4.79\times {10}^{-2}\right)$
Rural	1239.17	$1.84\times {10}^{6}\left(2.40\times {10}^{5}-5.20\times {10}^{6}\right)$	$1.48\times {10}^{-1}\left(1.94\times {10}^{-2}-4.20\times {10}^{-1}\right)$	$2.76\times {10}^{5}\left(1.43\times {10}^{4}-9.88\times {10}^{5}\right)$	$2.22\times {10}^{-2}\left(1.16\times {10}^{-3}-7.97\times {10}^{-2}\right)$
Immunocompetent	2970.00	$2.09\times {10}^{6}\left(2.51\times {10}^{5}-6.32\times {10}^{6}\right)$	$7.05\times {10}^{-2}\left(8.45\times {10}^{-3}-2.13\times {10}^{-1}\right)$	$2.96\times {10}^{5}\left(1.56\times {10}^{4}-1.06\times {10}^{6}\right)$	$9.96\times {10}^{-3}\left(5.26\times {10}^{-4}-3.57\times {10}^{-2}\right)$
Immunodeficient	1.63	$3.18\times {10}^{3}\left(4.20\times {10}^{2}-7.90\times {10}^{3}\right)$	$1.95\times {10}^{-1}\left(2.57\times {10}^{-2}-4.48\times {10}^{-1}\right)$	$5.03\times {10}^{2}\left(3.23\times {10}^{1}-1.68\times {10}^{3}\right)$	$3.09\times {10}^{-2}\left(1.98\times {10}^{-3}-1.03\times {10}^{-1}\right)$
Surface water	2285.13	$2.01\times {10}^{6}\left(2.39\times {10}^{5}-5.90\times {10}^{6}\right)$	$8.80\times {10}^{-2}\left(4.04\times {10}^{-2}-2.58\times {10}^{-1}\right)$	$2.94\times {10}^{5}\left(1.29\times {10}^{4}-1.06\times {10}^{6}\right)$	$1.29\times {10}^{-2}\left(5.66\times {10}^{-4}-4.62\times {10}^{-2}\right)$
Groundwater	686.50	$1.54\times {10}^{4}\left(7.29\times {10}^{2}-5.71\times {10}^{4}\right)$	$2.24\times {10}^{-3}\left(1.06\times {10}^{-4}-8.31\times {10}^{-3}\right)$	$5.83\times {10}^{3}\left(1.41\times {10}^{2}-2.25\times {10}^{4}\right)$	$8.50\times {10}^{-4}\left(2.06\times {10}^{-5}-3.28\times {10}^{-3}\right)$
Piped supply	2091.82	$2.54\times {10}^{5}\left(1.34\times {10}^{4}-9.38\times {10}^{5}\right)$	$1.21\times {10}^{-2}\left(6.39\times {10}^{-4}-4.48\times {10}^{-2}\right)$	$2.71\times {10}^{4}\left(8.58\times {10}^{2}-1.05\times {10}^{5}\right)$	$1.29\times {10}^{-3}\left(4.10\times {10}^{-5}-5.04\times {10}^{-3}\right)$
Direct supply	879.81	$2.00\times {10}^{6}\left(2.55\times {10}^{5}-5.81\times {10}^{6}\right)$	$2.28\times {10}^{-1}\left(2.89\times {10}^{-2}-6.60\times {10}^{-1}\right)$	$2.95\times {10}^{5}\left(1.57\times {10}^{4}-1.08\times {10}^{6}\right)$	$3.36\times {10}^{-2}\left(1.79\times {10}^{-3}-1.22\times {10}^{-1}\right)$
Total	2971.63	$2.02\times {10}^{6}\left(2.50\times {10}^{5}-5.91\times {10}^{6}\right)$	$6.81\times {10}^{-2}\left(8.41\times {10}^{-3}-1.99\times {10}^{-1}\right)$	$2.96\times {10}^{5}\left(1.57\times {10}^{4}-1.06\times {10}^{6}\right)$	$9.96\times {10}^{-3}\left(5.28\times {10}^{-4}-3.57\times {10}^{-2}\right)$

^a^*95% CI*: 95% confidence intervals were based on the results from 10,000 model iterations

### Health burden by population group and water source

**Fig. 3:**
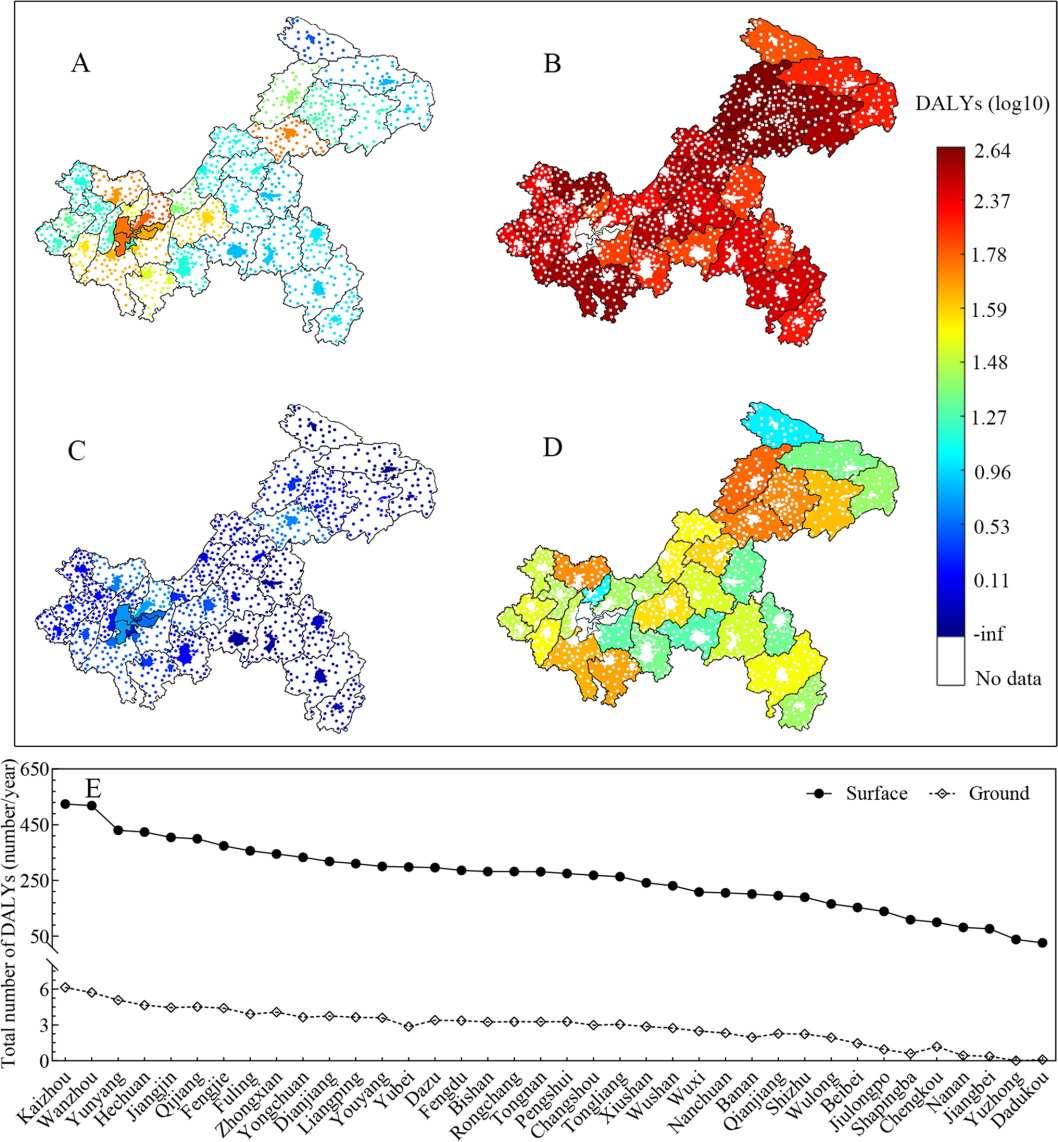
Estimated annual disease burden (in DALYs) attributable to *Cryptosporidium* and *Giardia* consumed via surface and ground waters in urban and rural areas for each Chongqing district or county. **A** Urban *Cryptosporidium* DALYs, **B** rural *Cryptosporidium* DALYs, **C** urban *Giardia* DALYs, **D** rural *Giardia* DALYs, and **E** total DALYs attributable to *Cryptosporidium* and *Giardia*

The estimated total health burden from *Cryptosporidium* and *Giardia* infections associated with three exposure routes in surface and ground water was $2.96\times {10}^{-4}$ DALYs $\left(95\% \mathrm{CI}:3.80\times {10}^{-5} \mathrm{to} 8.29\times {10}^{-4}\right)$ and $3.96\times {10}^{-5}$ DALYs $\left(95\% \mathrm{CI}:2.17\times {10}^{-6} \mathrm{to} 1.38\times {10}^{-4}\right)$, respectively (Table 3). The health burden for immunodeficiency was much higher than for immunocompetence. Total DALYs caused by *Cryptosporidium* and *Giardia* ranged from 1 to ${10}^{2}$ in urban areas (Fig. 3A, C) and from ${0}$ to ${10}^{3}$ in rural areas (Fig. 3B, D). The highest total DALYs were in areas of large population, such as the main urban districts of Yubei, Jiulongpo, and Shapingba (Fig. 3A, C), and the rural areas of Kaizhou, Wanzhou, and Yunyang (Fig. 3B, D). Total DALYs caused by *Cryptosporidium* and *Giardia* ranged from ${10}$ to ${10}^{3}$ for surface water and ${0}$ to ${10}$ for groundwater (Fig. 3E). The breakdown by group in Table 3 demonstrates that the disease burdens caused by *Cryptosporidium* and *Giardia* in the TGR watershed resulted from direct consumption of source water, especially surface water, and affected males more than females.

**Table 3:** Simulated disease burden (in DALYs) caused by *Cryptosporidium* and *Giardia* by exposure routes and subgroups in the TGR watershed, and DALYs per person per year (pppy) per group

		*Cryptosporidium*		*Giardia*	
Exposure route	Total population in group $\times {10}^{4}$	Total number of DALYs (95% CI^a^)	DALYs/pppy in group (95% CI)	Total number of DALYs (95% CI)	DALYs/pppy in group (95% CI)
Incidental intake	2971.63	$7.31\times {10}^{3}\left(5.97\times {10}^{2}-2.19\times {10}^{4}\right)$	$2.55\times {10}^{-4}\left(2.17\times {10}^{-5}-7.73\times {10}^{-4}\right)$	$7.87\times {10}^{2}\left(2.95\times {10}^{1}-3.11\times {10}^{3}\right)$	$2.72\times {10}^{-5}\left(1.03\times {10}^{-6}-1.05\times {10}^{-4}\right)$
Direct drinking	2971.63	$1.38\times {10}^{3}\left(4.75\times {10}^{2}-2.47\times {10}^{3}\right)$	$4.80\times {10}^{-5}\left(1.66\times {10}^{-5}-8.64\times {10}^{-5}\right)$	$3.64\times {10}^{2}\left(3.09\times {10}^{1}-9.95\times {10}^{2}\right)$	$1.26\times {10}^{-5}\left(1.13\times {10}^{-6}-3.45\times {10}^{-5}\right)$
Swimming	2971.63	$3.28\times {10}^{2}\left(1.73\times {10}^{1}-1.21\times {10}^{3}\right)$	$1.15\times {10}^{-5}\left(6.13\times {10}^{-7}-4.18\times {10}^{-5}\right)$	$3.2\times {10}^{1}\left(1.04-1.30\times {10}^{2}\right)$	$1.10\times {10}^{-6}\left(3.60\times {10}^{-8}-4.31\times {10}^{-6}\right)$
Group					
Urban	1732.46	$1.00\times {10}^{3}\left(4.72\times {10}^{1}-3.70\times {10}^{3}\right)$	$5.77\times {10}^{-5}\left(2.72\times {10}^{-6}-2.14\times {10}^{-4}\right)$	$8.09\times {10}^{1}\left(2.13-3.31\times {10}^{2}\right)$	$4.63\times {10}^{-6}\left(1.19\times {10}^{-7}-1.81\times {10}^{-5}\right)$
Rural	1239.17	$7.88\times {10}^{3}\left(1.06\times {10}^{3}-2.17\times {10}^{4}\right)$	$6.36\times {10}^{-4}\left(8.53\times {10}^{-5}-1.75\times {10}^{-3}\right)$	$1.08\times {10}^{3}\left(5.82\times {10}^{1}-3.74\times {10}^{3}\right)$	$8.84\times {10}^{-5}\left(4.72\times {10}^{-6}-3.10\times {10}^{-4}\right)$
Immunocompetent	2970.00	$4.71\times {10}^{3}\left(5.30\times {10}^{2}-1.44\times {10}^{4}\right)$	$1.59\times {10}^{-4}\left(1.79\times {10}^{-5}-4.86\times {10}^{-4}\right)$	$4.68\times {10}^{2}\left(2.17\times {10}^{1}-1.70\times {10}^{3}\right)$	$1.58\times {10}^{-5}\left(7.33\times {10}^{-7}-5.75\times {10}^{-5}\right)$
Immunodeficient	1.63	$4.28\times {10}^{3}\left(4.08\times {10}^{2}-1.27\times {10}^{4}\right)$	$2.62\times {10}^{-1}\left(2.50\times {10}^{-2}-7.81\times {10}^{-1}\right)$	$6.99\times {10}^{2}\left(3.31\times {10}^{1}-2.47\times {10}^{3}\right)$	$4.35\times {10}^{-2}\left(2.04\times {10}^{-3}-1.59\times {10}^{-1}\right)$
Surface water	2285.13	$8.78\times {10}^{3}\left(1.07\times {10}^{3}-2.46\times {10}^{4}\right)$	$3.84\times {10}^{-4}\left(4.66\times {10}^{-5}-1.07\times {10}^{-3}\right)$	$1.15\times {10}^{3}\left(5.55\times {10}^{1}-2.47\times {10}^{3}\right)$	$5.10\times {10}^{-5}\left(2.37\times {10}^{-6}-1.84\times {10}^{-4}\right)$
Groundwater	686.50	$8.72\times {10}^{1}\left(3.32-3.46\times {10}^{2}\right)$	$1.27\times {10}^{-5}\left(4.84\times {10}^{-7}-5.04\times {10}^{-5}\right)$	$2.50\times {10}^{1}\left(5.30\times {10}^{1}-9.79\times {10}^{1}\right)$	$3.53\times {10}^{-6}\left(7.70\times {10}^{-8}-1.37\times {10}^{-5}\right)$
Piped supply	2091.82	$9.10\times {10}^{2}\left(4.51\times {10}^{1}-3.25\times {10}^{3}\right)$	$4.35\times {10}^{-5}\left(2.15\times {10}^{-6}-1.55\times {10}^{-4}\right)$	$6.67\times {10}^{1}\left(1.94-2.73\times {10}^{2}\right)$	$3.29\times {10}^{-6}\left(9.40\times {10}^{-8}-1.32\times {10}^{-5}\right)$
Direct supply	879.81	$7.77\times {10}^{3}\left(1.00\times {10}^{3}-2.13\times {10}^{4}\right)$	$8.84\times {10}^{-4}\left(1.14\times {10}^{-4}-2.42\times {10}^{-3}\right)$	$1.07\times {10}^{3}\left(5.93\times {10}^{1}-3.80\times {10}^{3}\right)$	$1.22\times {10}^{-4}\left(6.41\times {10}^{-6}-4.33\times {10}^{-4}\right)$
Male	1532.71	$5.34\times {10}^{3}\left(6.42\times {10}^{2}-1.48\times {10}^{4}\right)$	$3.48\times {10}^{-4}\left(4.19\times {10}^{-5}-9.65\times {10}^{-4}\right)$	$7.30\times {10}^{2}\left(3.98\times {10}^{1}-2.52\times {10}^{3}\right)$	$4.76\times {10}^{-5}\left(2.59\times {10}^{-6}-1.64\times {10}^{-4}\right)$
Female	1438.92	$3.49\times {10}^{3}\left(4.24\times {10}^{2}-9.67\times {10}^{3}\right)$	$2.43\times {10}^{-4}\left(2.95\times {10}^{-5}-6.72\times {10}^{-4}\right)$	$4.30\times {10}^{2}\left(2.35\times {10}^{1}-1.51\times {10}^{3}\right)$	$2.99\times {10}^{-5}\left(1.63\times {10}^{-6}-1.05\times {10}^{-4}\right)$
Total	2971.63	$8.80\times {10}^{3}\left(1.13\times {10}^{3}-2.46\times {10}^{4}\right)$	$2.96\times {10}^{-4}\left(3.80\times {10}^{-5}-8.29\times {10}^{-4}\right)$	$1.16\times {10}^{3}\left(6.29\times {10}^{1}-4.02\times {10}^{3}\right)$	$3.96\times {10}^{-5}\left(2.17\times {10}^{-6}-1.38\times {10}^{-4}\right)$

^a^*95% CI*: 95% confidence intervals were based on the results from 10,000 model iterations

**Fig. 4:**
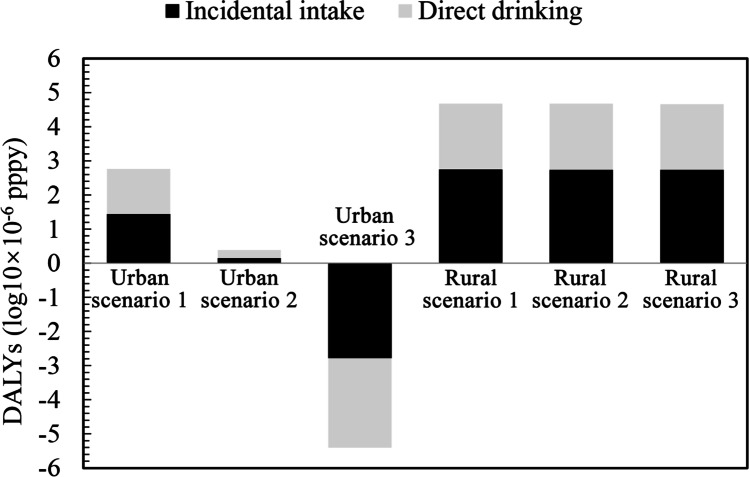
Simulated disease burden (in DALYs) caused by *Cryptosporidium* in drinking water by region, exposure route, and type of water treatment. Scenario 1: conventional treatment, scenario 2: advanced treatment using ozone, and scenario 3: advanced treatment using ozone and microfiltration

Ozone and microfiltration were compared with conventional water treatment, taking *Cryptosporidium* as a representative contaminant. The total health burden from drinking water (incidental and direct intake) was $3.13\times {10}^{-6}$ DALYs $\left(95\% \mathrm{CI}:2.74\times {10}^{-11} \mathrm{to} 1.68\times {10}^{-5}\right)$ following ozone treatment and $4.01\times {10}^{-9}$ DALYs $\left(95\% \mathrm{CI}:1.31\times {10}^{-13} \mathrm{to} 2.48\times {10}^{-8}\right)$ following ozone and microfiltration treatment in urban areas, and $6.25\times {10}^{-4}$ DALYs $\left(95\% \mathrm{CI}:8.77\times {10}^{-5} \mathrm{to} 1.73\times {10}^{-3}\right)$ for both treatments in rural areas (Fig. 4). The risk to the urban population consuming tap water treated with ozone and microfiltration would be considered acceptable as it is much lower than the level of acceptable waterborne exposure risk of ${10}^{-{6}}$ DALYs pppy recommended by the World Health Organization (WHO 2011).

**Fig. 5:**
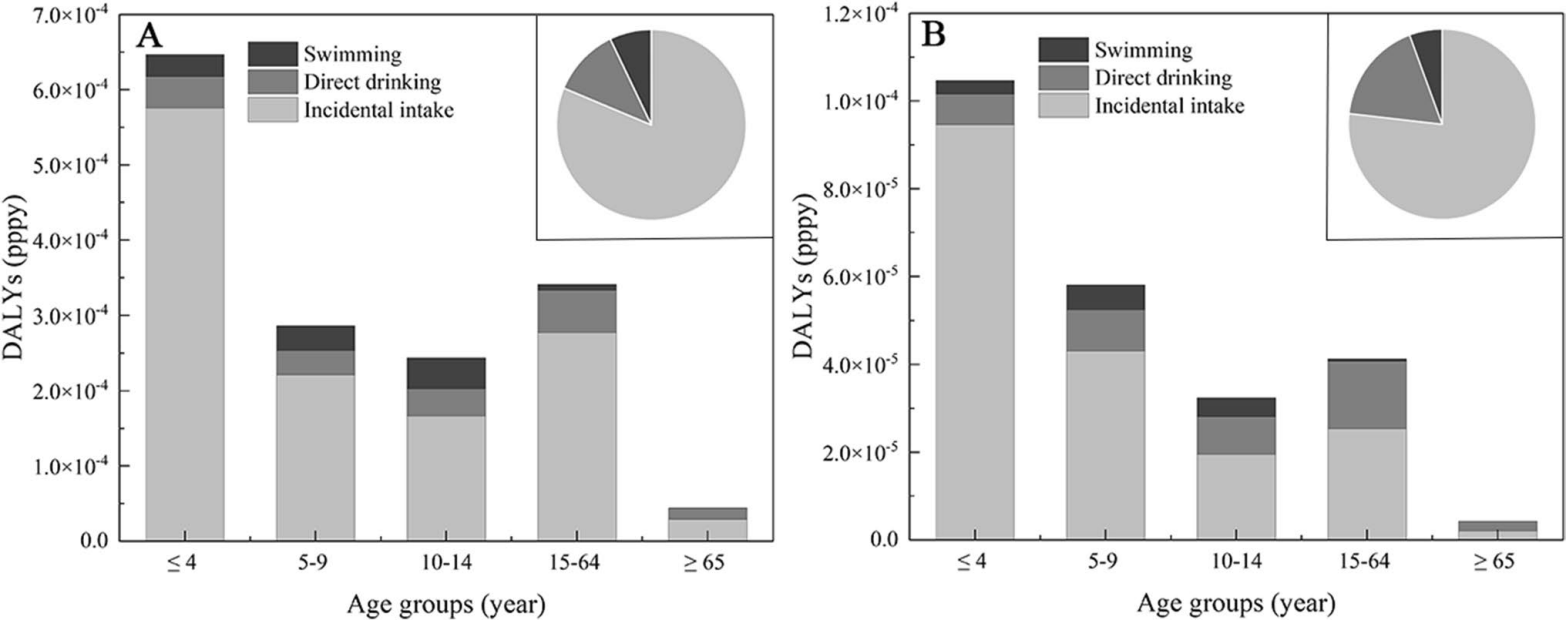
The disease burden in DALYs (pppy) attributable to *Cryptosporidium* (**A**) and *Giardia* (**B**) for different exposure routes and ages in Chongqing. The histograms illustrate the disease burden from all three exposure routes across different age groups, and the pie charts show the cumulative burden of the three routes across all ages

Figure 5 illustrates the cryptosporidiosis and giardiasis burden based on exposure route and age group. Incidental intake accounted for 83.5% and 77.0% of the cumulative burden for *Cryptosporidium* and *Giardia*, while swimming intake accounted for less than 7.3% and 5.7%, respectively. Among the age groups, the burden of protozoal disease was highest in children (≤ 4 years) at the individual level, while the lowest was associated with the elderly (≥ 65 years) (Fig. 5). The highest DALYs due to *Cryptosporidium* and *Giardia* in the immunocompetent population were associated with outpatient cases (39%), followed by mild (28%), fatal (21%), and hospitalized cases (12%) (Fig. S1). For the immunodeficient, the highest total DALYs were associated with fatal cases.

### Sensitivity analysis

A sensitivity analysis was performed by measuring Spearman’s rank order correlation to study the effects of the variation of model inputs on the final output of protozoa concentrations and disease burden. Protozoa concentrations in surface water were most sensitive to removal efficiency (${{F}}_{\mathrm{rem}}$) and cryptosporidiosis/giardiasis prevalence (*P*_*h*_) (Fig. S2). Protozoa concentrations in groundwater were most sensitive to depth of groundwater (${{D}}_{\mathrm{ground}}$) and reduction factor for transport in soil (${{f}}_{\mathrm{soil}}$) (Fig. S3). The most sensitive input parameters when assessing the health burden imposed by surface and ground waters were the concentration of *Cryptosporidium* in the TGR (${{C}}_{\mathrm{s,} \, {\text{Crypt}}\mathrm{.}}$), infection-enhancing coefficient of the synergistic effect of HIV on *Cryptosporidium* in rural areas (${{f}}_{\mathrm{HIV,} \, \text{Crypt.}\mathrm{, rural}}$), and infectivity constant for *Cryptosporidium* in rural areas (${{r}}_{\mathrm{Crypt.,} \, {\text{rural}}}$) (Fig. S4).

## Discussion

The Yangtze Three Gorges Project is one of the largest freshwater resources in the world but no information is available on the risk from pathogenic parasites consumed via surface and ground waters within its watershed. This study provides a new spatially explicit GloWPa-TGR-Crypt-Giar C1 model for the TGR watershed at Chongqing. It estimates *Cryptosporidium* and *Giardia* concentrations, pinpoints high concentration regions for further study, and explores the relative importance of pathogens contributing to the disease burden from consumption of surface and ground waters. This model can be applied to other bacteria, protozoa, and viruses and to other watersheds to evaluate water quality and health risks and to suggest effective mitigation measures.

Monthly mean concentrations of *Cryptosporidium* in the TGR reached 19.3 oocysts/10 L (mean 6.6 oocysts/10 L). *Giardia* reached 5.0 cysts/10 L (mean 1.8 cysts/10 L). Xiao et al. (2013) reported mean *Cryptosporidium* and *Giardia* in the TGR region at 1.9–6.3 oocysts/10 L and 0.8–7.6 cysts/10 L, respectively. Liu et al. (2019) used a SWAT model to simulate the Daning River watershed of the TGR. Mean annual *Cryptosporidium* concentration was 9.5 oocysts/10 L. Thus, (oo)cyst levels predicted by the present study are similar to previous reports. *Cryptosporidium* and *Giardia* monthly mean concentrations in groundwater ranged from 0.007 to 0.3 oocysts/10 L and 0.002 to 0.2 cysts/10 L, respectively. They ranged from 0 to 0.66 oocysts/10 L and 0 to 0.05 cyst/10 L, respectively, in groundwater sampled in Germany (Gallas-Lindemann et al. 2013). *Cryptosporidium* (0–0.75 oocysts/10 L) and *Giardia* (0–0.76 cyst/10 L) were reported in tubewell water in a study of 60 Indian villages (Odagiri et al. 2016; Daniels et al. 2018), but there is no such information on Chinese groundwater. This is the first study to simulate monthly pathogen concentrations in groundwater in the TGR watershed at district/county level. (Oo)cyst concentrations in groundwater were slightly lower than previously reported, but the median predicted concentrations mostly fell within reported ranges. This may be due to the topography of Chongqing being predominantly (76%) hills and mountains (http://vdb3.soil.csdb.cn/) and a high runoff fraction (0.1–0.3 using Eq. 4C in Table S2) being applied to estimate the load ending up in rivers. By comparison, one model (Hofstra and Vermeulen 2016) applied a constant runoff fraction of 0.025, such that most of the pathogen remained on land and may have subsequently entered the groundwater. Future research should explore the causal link between topography and protozoa concentrations in surface and ground waters.

This is the first QMRA to explore *Cryptosporidium* and *Giardia* health risks from surface and ground waters in the TGR watershed. It encompasses multiple transmission pathways and shows that the microbial quality of the water is unsafe when consumed directly or following conventional treatment. Incidental intake causes the highest morbidity (Table 2) given that the fraction of the exposed population who drank unboiled tap water (4.5%) was much lower than those who ingested unboiled tap water via incidental intake (100%). Han et al. (2020) also reported that the highest risk of *Cryptosporidium* and *Giardia* infection in the water of 44 cities in China was from incidental intake. We estimated morbidity in urban areas to be 1172.9 cryptosporidiosis cases (95% CI: 62.3–4354.9) and 120.9 giardiasis cases (95% CI: 3.3–479.3) per 100,000 people per year. A similar result (1060.2 per 100,000 people/year for both diseases) has been reported in the TGR (exposure via incidental intake, direct drinking, and swimming) (Xiao et al. 2013). Xiao et al. (2012) reported 1.5 cryptosporidiosis cases per 100,000 immunocompetent people per year in China associated with conventionally treated drinking water (2.1 oocysts/10 L). The present study estimated higher morbidity because a higher oocyst concentration and intake via swimming were taken into account. Morbidity for immunodeficient people was 0.2 pppy (95% CI: 0.03–0.5) for *Cryptosporidium* and 0.03 pppy (95% CI: 0.002–0.1) and for *Giardia*. Similar *Cryptosporidium* morbidity (mean 0.2 pppy; 95% CI: 0.2–0.5) was estimated for consumption of surface water in sub-Saharan Africa (Limaheluw et al. 2019).

This study observed a tenfold greater incidence of disease in rural areas than in cities (Table 2). A similar result was reported in epidemiological investigations of *Cryptosporidium* in 27 Chinese provinces (Liu et al. 2020). The mean prevalence in Chongqing was 4.8% (3.4–8.0%), with infection rates of 0–3.7% in urban areas and 1.8–12.9% in rural areas. Similar results have been reported in other countries (Liu et al. 2020). From 2004 to 2011, New Zealand saw more cryptosporidiosis cases in rural areas (annual mean 71.5 cases per 100,000; 95% CI: 64.6–78.3) than urban areas (annual mean 29.2 cases per 100,000; 95% CI: 26.9–31.5) (Cowie and Bell 2013). *Cryptosporidium* disease burden from surface water in sub-Saharan Africa was also much greater in rural areas (30% of the population; 95% CI: 5–30) than urban areas (3%; 95% CI: 0.9–7.0) (Limaheluw et al. 2019). Infectious diseases often disproportionately affect rural populations due to the poorer sanitation conditions, lack of general health knowledge, and health habits in many rural areas of less developed countries (Liu et al. 2020). Daniels et al. (2018) highlighted that diarrheal disease burdens in rural areas may persist despite improvements in sanitation and hygiene, unless drinking water is made safe and reliable at the source or through effective household water treatment. Further scenario analyses are required to elucidate the impact of these factors on the health burden.

Unsafe sanitation or the unsafe management of excreta discharged into the environment lead to surface and groundwater contamination and exposure to pathogens (Mraz et al. 2021). The cumulative disease burden from *Cryptosporidium* and *Giardia* at Chongqing ($4.45\times {10}^{-4}$ DALYs pppy from surface water, $1.62\times {10}^{-5}$ from groundwater) is higher than the WHO’s recommended maximum waterborne exposure risk of $1.0\times {10}^{-6}$ DALYs pppy (WHO 2011). The Global Burden of Disease Study 2017 estimated the burden of diarrheal disease in China across all ages to be up to $3.05\times {10}^{-4}$ DALYs pppy $\left(95\% \mathrm{CI}:2.60-3.55\times {10}^{-4}\right)$ (GBD Diarrhoeal Diseases Collaborators 2017). This means that the risk from surface water-associated cryptosporidiosis and giardiasis in the TGR Chongqing region is greater than diarrheal disease risk in China as a whole. Epidemiological investigations in 27 provincial administrative regions in China also showed that 4.8% (3.4–8.0%) of people in Chongqing were diagnosed as having a *Cryptosporidium* infection or cryptosporidiosis, which exceeds the average prevalence in China (3.0%, ranging from 0.65 to 11.15%) (Liu et al. 2020).

Gravitational percolation through pores in the soil matrix and/or bedrock fractures is the most frequently reported ingress mechanism for protozoal contamination of groundwater (Chique et al. 2020). Murphy et al. (2017) estimated an annual global burden of 35.2–59.4 million cases of gastrointestinal disease linked to groundwater sources, with *Cryptosporidium* being identified as the etiological agent in seven outbreaks between 1948 and 2016. This is probably an underestimate due to the lack of surveillance. It is plausible that protozoa in rural groundwater drinking sources (even at low concentrations) may account for a significant portion of the disease burden in settings were tubewells are used for drinking water. Such sources should be systematically monitored (Daniels et al. 2018).

The majority of cases in this study occurred in people directly consuming surface water. Simulated *Cryptosporidium* burden in drinking water subjected to three different treatments indicated that the risk to people consuming tap water following ozone and microfiltration treatment was acceptable in urban areas (< ${10}^{-{6}}$DALYs), but unacceptable in rural areas where only 29% of consumed water was piped and treated. Improving sanitation through enhanced water treatment should be considered when attempting to reduce disease burden. It is vital that water, sanitation, and hygiene practitioners advocate and implement appropriate treatment and management systems for the removal of pathogens if we truly want to reduce disease burdens and achieve the United Nations Sustainable Development Goal 6 target of safely managed water and sanitation (Mraz et al. 2021).

Immunodeficient consumers in Chongqing carried nearly 50% of the total cryptosporidiosis and giardiasis health burden. This is extremely high considering they are a very small fraction of the population (0.056%) (Office 2008). Cryptosporidiosis cases were 4.2 times and giardiasis cases 2.4 times higher in immunodeficient people than in the immunocompetent (Han et al. 2020), and mortality in the former subpopulation was much higher than in the latter (Xiao et al. 2012). Furthermore, immunocompromised children (≤ 4 years) carried a higher burden than adults. Globally, diarrhea DALYs for children under 5 years ($1.48\times {10}^{-3}$ DALYs pppy) are nearly fivefold higher than for all ages ($3.1\times {10}^{4}$ DALYs pppy) (Khalil et al. 2018). Diarrhea and *Cryptosporidium* or *Giardia* infections impair growth causing long-term health problems (e.g., low weight) and having immediate, acute impacts on systemic and mucosal immune system functions in young children (Ajjampur et al. 2011; Khalil et al. 2018). This study highlights the significance of immune status as a risk factor for these infections and disease burdens. Other population groups, such as cancer patients, have a high incidence of *Cryptosporidium* (47.8% in China), as they often experience transient or continuous impairment of immunity due to treatments such as chemotherapy (Liu et al. 2020). It needs to be established if there is a causal link between cancer and waterborne pathogen infections and, if so, to quantify the associated additional burden.

The health risk of cryptosporidiosis or giardiasis was higher for males than females. This can be attributed to the high proportion (71.3%) of the HIV-positive subpopulation in China being male (Office 2008). The disease burden in the immunocompetent population was primarily on outpatient cases, followed by mild, fatal, and hospitalized cases. Similar results were reported at the recreational lakes in Tianjin, China (Xiao et al. 2018b). The highest disease burden in the immunodeficient population fell on fatal cases, with the case fatality ratio being 12,000 times higher than in the immunocompetent population (Xiao et al. 2012).

Sensitivity analysis showed that simulated protozoa concentrations were most influenced by removal efficiency, pathogen prevalence in humans, and groundwater depth and that protozoa concentrations influenced disease burden. The high concentrations predicted in hotspot regions are a concern because of the large populations with insufficient sanitation (Hofstra and Vermeulen 2016; Huang et al. 2020). Pathogenic contamination of source waters due to inadequate sanitation will be influenced by population growth, urbanization, and climate change, which will, in turn, impact the exposure dose–response (Hofstra et al. 2019; Okaali et al. 2021). Additional observational data will improve this modeling (Okaali et al. 2021). Future research should explore process-based modeling and scenario analysis based on socio-economic pathways and representative concentration pathways for waterborne pathogens and the consequent diarrheal disease burden. This will lead to a better understanding of the impacts of environmental change and management scenarios on disease burden and support management decisions by identifying the most effective control measures.

## Conclusion

- This new GloWPa-TGR-Crypt-Giar C1 model simulates *Cryptosporidium* and *Giardia* concentrations arising from point and nonpoint sources in surface and ground waters at district/county level in the Chongqing TGR watershed area.
- (Oo)cyst concentrations were predicted to be 100-fold higher in surface water than groundwater in most regions.
- The cumulative disease burden was much higher in rural areas than urban areas, and much higher from surface water than groundwater (all exceeding the threshold recommended by the WHO). Most disease burden was a consequence of direct supply.
- A substantial disease burden was carried by immunodeficiency and children under 4 years.
- Consequently, environmental policymakers should pay more attention to water supply patterns, sensitive subpopulations, and improvement of sanitation via effective water treatment to reduce disease burdens.
- The approach used in this modeling could be applied to other bacteria, protozoa, and viruses, and to other watersheds to evaluate water quality and associated health risks in order to develop more effective regulation and control strategies.

## Supplementary Information

Below is the link to the electronic supplementary material.

Supplementary file1 (PDF 1222 kb)

## Data Availability

All data generated or analyzed during this study are included in this published article.
