# Supplementary material for: Spatially explicit model of the Cryptosporidium and Giardia disease burden from surface and ground waters in urban and rural areas of the Three Gorges Reservoir watershed in Chongqing, China
Source: Environ Sci Pollut Res Int. 2022 Dec 26;30(13):37127–42. doi: 10.1007/s11356-022-24690-2 (PMC10039849; doi:10.1007/s11356-022-24690-2)
Supplement: Supplementary file 1 — Supplementary file1 (PDF 1222 kb) [file 11356_2022_24690_MOESM1_ESM.pdf]

Spatially explicit model of the *Cryptosporidium* and *Giardia* disease burden from surface and ground waters in urban and rural areas of the Three Gorges Reservoir watershed in Chongqing, China

Qian Huang[1], Shan Huang[2], Bo Li[1], Yanhong Xiong[1], Weijie Kuang[1], Shunxin Xiao[1], Jianghui Yi[1], Feng Zhao[1], Guosheng Xiao[1,*]

[1] College of Biology and Food Engineering, Chongqing Three Gorges University, Wanzhou, 404130, China

[2] College of Grassland Science and Technology, Sichuan Agricultural University, Chengdu, 611130, China

* **Correspondence**: xgs03@sanxiau.edu.cn, Tel.: +86–023–58108213

Table S1 Chongqing population in different age groups, life expectancy and their sensitivity parameters with *Cryptosporidium* and *Giardia* infection.

Years	Urban population ($\times 10^4$)[a]		Urban immunodeficiency subpopulation($\times 10^2$)[b]		Rural population ($\times 10^4$)		Rural immunodeficiency subpopulation($\times 10^2$)		Life expectancy (e_j)[c]		Sensitivity parameters (S_j)[d]	
	Male	Female	Male	Female	Male	Female	Male	Female	Male	Female	*Crypt.*	*Giar.*
≤ 4	50.34	47.28	0.45	0.20	36.00	33.82	0.31	0.14	73.64	79.43	10.59	17.91
5-9	47.58	44.69	0.42	0.19	34.03	31.97	0.30	0.13	73.10	78.74	1.71	5.96
10-14	48.51	45.57	0.74	0.32	34.70	32.59	0.53	0.23	69.40	74.92	0.99	1.88
15-64	640.37	601.46	65.08	24.18	458.04	430.21	46.55	17.29	43.06	48.06	1.000	1.00
≥ 65	106.08	99.63	2.56	0.93	75.87	71.26	1.83	0.67	10.11	21.52	0.20	0.26

[a]The population were based on Chong Qing statistical yearbook 2014 (http://tjj.cq.gov.cn/tjsj/shuju/tjnj/).
[b]The immunodeficiency subpopulation were based on literature (Office 2008).
[c]The data were based on National Bureau of Statistics of China. (2017) China statistical yearbook 2016 (http://www.stats.gov.cn/tjsj/ndsj/2016/indexeh.htm).
[d]The sensitivity parameters were based on literature (Han et al. 2020)

Section 1: Calculating sources of *Cryptosporidium* and *Giardia* loads

The GloWPa-TGR-Crypt-Giar C1model calculated point source loads to the TGR by multiplying population (P_u, P_r), annual (oo)cyst excretion per person (O_p), and removal efficiency depending on the sanitation systems (F_{rem}) (**Eq. 1A** and **1B**, Table S2). Compared to the earlier work, GloWPa-TGR-Crypt-Giar C1 calculated annual (oo)cyst excretion per person by multiplying annual feces per person (M_h), daily (oo)cyst excretion rate per infected person (O_h), cryptosporidiosis and giardiasis prevalence (P_h) (**Eq.2**, Table S2). The model calculated non-point source loads on land was divided into two parts: rural residents (by multiplying population (P_r), annual feces (M_h), (oo)cyst excretion rate for infected person (O_h) and pathogen prevalence (P_h) as shown in **Eq. 1** (Table S2)), and livestock (by multiplying population (N_a), breeding days (D_a), daily manure (M_a), pathogen prevalence in livestock (O_a) and (oo)cyst excretion rate per infected livestock (P_a), respectively as shown in **Eq. 3** (Table S2)). We calculated nine livestock categories: cattle, sheep, goats, pigs, laying hens, broilers, ducks, goose and rabbits based on data for the year 2013 and 2016. Fece loads on land during irrigation after storage and temperature-dependent (oo)cyst decay during storage were accounted for in the model (Huang et al. 2020). Then, (oo)cysts transported from land to surface water via runoff (**Eq.4**, Table S2) and to groundwater via filtration (**Eq.5**, Table S2) (Fig. 1).

Table S2 Formulas and parameters for calculating (oo)cyst loads and concentrations in the TGR. watershed in Chongqing.

No Eq	Model description	Calculation formula	Parameter description	Parameter values	Reference
1A	Calculation of (oo)cyst loads from humans	$\text{st.}\begin{cases} K_1=CE_u=P_u\times F_{cu}\times O_p\times(1\text{-}F_{rem}) \\ K_2=CE_r=P_r\times F_{cr}\times O_p\times(1\text{-}F_{rem}) \\ K_3=DE_u=P_u\times F_{du}\times O_p \\ K_4=DifE_r=P_r\times F_{difr}\times O_p \end{cases}$	H: total (oo)cyst loads from human ((oo)cysts/year); P_u and P_r : total urban and rural populations in districts or counties, respectively; F_{cu}, F_{cr}, F_{du} and F_{difr}: the fractions of urban connected emissions, rural connected emissions, urban direct emissions and rural diffuse emissions, respectively (%); O_p: annual (oo)cyst excretion per person ((oo)cysts/year); F_{rem}: removal efficiency by sewage treatment plants (STP) (%).	$F_{cu:}$ was 77.99; $F_{du:}$ was 21.22; $F_{cr:}$ was 9; $F_{difr:}$ 91; F_{rem}: in Table S4.	(Chong Qing statistical yearbook 2014 and 2017 (http://tjj.cq.gov.cn/tjsj/shuju/tjnj/); 2013 China Environmental Status Bulletin (http://www.mee.gov.cn/hjzl/zghjzkgb/lnzghjzkgb/); 2013 China Environmental Status Bulletin (http://www.mee.gov.cn/hjzl/zghjzkgb/lnzghjzkgb/); Sun et al. 2014; Huang et al. 2020)
1B		$H=\sum_{i=1}^{4}K_i$			

No Eq	Model description	Calculation formula	Parameter description	Parameter values	Reference
2	Annual (oo)cyst excretion per person	$O_p = M_h \times O_h \times P_h$	O_p: annual (oo)cyst excretion per person ((oo)cysts/year);<br>M_h: annual feces per person (kg day^{-1});<br>O_h: daily (oo)cyst excretion rate per infected person ((oo)cysts/ kg);<br>P_h: pathogens prevalence in human (%).	Values summarized in Tables S8-10.	(Vermeulen et al. 2017)
3	Calculation of (oo)cyst loads from animals	$A = \sum_{j=1}^{9} N_{a_j} \times D_{a_j} \times M_{a_j} \times O_{a_j} \times P_{a_j} \times S_a$	*A:* total (oo)cyst loads from animals ((oo)cysts/year);<br>N_a: animal population at categories *j*;<br>D_a: breeding days at categories *j* (days);<br>M_a: daily manure at categories *j* (kg day^{-1});<br>O_a: (oo)cyst excretion rate per infected livestock at categories *j* ((oo)cysts/ kg);<br>P_a: pathogens prevalence in livestock at categories *j* (%).<br>S_a: species of *Cryptosporidium* and *Giardia* in animal.	Values summarized in Tables S4, 8-11.	(Chong Qing statistical yearbook 2014 and 2017 (http://tjj.cq.gov.cn/tjsj/shuju/tjnj/); Huang et al. 2020)
4A<br><br>4B	Calculation of (oo)cyst load to the TGR	$E_{TGR,\,m} = CE_{u,m} + CE_{r,m} + DE_{u,m} + (DifE_{r,m} \times F_{s,h} \times F_{v,m} + A \times F_{s,a} \times F_{v,m}) \times F_{run,m}$<br><br>$F_{v,m} = \frac{\int_0^{t_s} e^{-\left(-\frac{\ln 10}{-2.5586 \times T_m + 119.63_s} \times t_s\right)} dt}{t_s}$	$E_{TGR,\,m}$: (oo)cyst load to the TGR in month *m* ((oo)cysts /month);<br>$CE_{u,m}$, $CE_{r,m}$, $DE_{u,m}$ *and* $DifE_{r,m}$: urban	$F_{s,h}$ and $F_{s,a}$: was in Table S4;<br>t_s: was in Table S4;<br>T_m: was in Table	(China Soil Database (http://vdb3.soil.csdb.cn/); Velthof et

No Eq	Model description	Calculation formula	Parameter description	Parameter values	Reference
4C		$F_{run,m} = F_{run,max} \times f_{lu} \times f_{p,m} \times f_{rc} \times f_s$	connected emissions, rural connected emissions, urban direct emissions and rural diffuse emissions , in month *m* respectively ((oo)cysts /month);<br>$F_{s,h}$ and $F_{s,a}$: proportions of stored manure applied as a fertilizer from rural residents and livestock, respectively;<br>$F_{v,m}$: proportion of (oo)cyst survival in the storage system in month *m*;<br>$F_{run,m}$: fraction of surface runoff in month *m*;<br>T_m: average air temperature in month *m* (℃);<br>t_s: manure storage time (days);<br>$F_{run,\ max}$: the fraction of maximum surface runoff across different slope classes;<br>f_{lu}: reduction factor for land use;<br>$f_{p,m}$: reduction factor for average monthly precipitation in month *m*;<br>f_{rc}: reduction factor for rock depth;<br>f_s: reduction factor for soil type.	S13;<br>$F_{run,\ max}$: was 0.5;<br>f_{lu}:was 1.0;<br>$f_{p,m}$:was in Table S3;<br>f_{rc}: was 0.8;<br>f_s: was 1.0.	al. 2009; Huang et al. 2020; Vermeulen et al. 2017)

No Eq	Model description	Calculation formula	Parameter description	Parameter values	Reference
5	Calculation of (oo)cyst loads on soil	$E_{soil,\ m}=(H_m+A_m)-E_{TGR,\ m}$	$E_{soil,\ m}$: (oo)cyst loads on soil in month *m* ((oo)cysts /month);<br>H_m: (oo)cyst loads from human in month *m* ((oo)cysts /month);<br>A_m: (oo)cyst loads from animals in month *m* ((oo)cysts /month);<br>$E_{TGR,\ m}$: (oo)cyst loads to the TGR in month *m* ((oo)cysts /month).		(Huang et al. 2020; Vermeulen et al. 2017)
6	Total inactivation rate	$K_m=K_4e^{\lambda(T_{w,m}-4)}+\frac{k_l\ I_{A,m}}{k_d\ C_{DOC,m}\ Z_m}\ \times(1-e^{-k_dC_{DOC,m}Z_m})+\frac{v}{Z_m}$	K_m: total inactivation rate in month *m*;<br>K_4: decay rate constants at 4ºC;<br>λ: dimensionless modifier of temperature;<br>$T_{w,m}$: water temperature in month *m* (ºC);<br>k_l and k_d represent proportionality constants;<br>$I_{A,m}$: surface solar radiation in month *m* ($KJ^{-2}day^{-1}$);<br>$C_{DOC,m}$: dissolved organic carbon concentration in month *m* ($mg\ L^{-1}$) ;<br>Z_m: water level in month *m* (m);<br>v: settling velocity ($mday^{-1}$).	K_4: was 0.0051;<br>λ: was 0.18;<br>$T_{w,m}$: was in Table S13;<br>k_l: was 0.0004798;<br>k_d: was 9.831;<br>$I_{A,m}$: was in Table S14;<br>$C_{DOC,m}$: was in Table S14;<br>Z_m: was in Table S13;<br>v: 0.1.	(Vermeulen et al. 2019; Peng et al. 2000)

No Eq	Model description	Calculation formula	Parameter description	Parameter values	Reference
7	Transport time in the TGR	$t_{r,\,m}=\frac{L\times Z_m\times 1.22{Q_m}^{0.557}}{Q_m}$	$t_{r,\,m}$: residence time of (oo)cyst in Chongqing section of the TGR in month *m* (days);<br>L: length of the TGR in Chongqing section (m);<br>Z_m: water level in month *m* (m);<br>Q_m: reservoir inflow discharge in month *m* ($m^3 s^{-1}$).	*L:was 665;*<br>Q_m: was in Table S3.	(Vermeulen et al. 2019; Cheng et al. 2019)

Table S3 The statistics of monthly reservoir inflow discharge and precipitation during the year 2009-2012 in Chongqing section of the TGR.

Month	Reservoir inflow discharge(Q_m) [a]		Precipitation (P_m) [b]		Reduction factor ($f_{p,m}$)
	Mean value ($m^3 s^{-1}$)	Probability distribution	Mean value(mm)	Probability distribution	
Jan.	4866	Gamma (31.39, 155.04)	10.63	Lognormal (10.632, 7.4181)	0.25
Feb.	4150	Gamma (765.11, 5.424)	17.84	BetaGeneral (2.4818, 9.4444, 0, 85.747)	0.25
Mar.	4094	Gamma (14.144, 289.48)	42.31	BetaGeneral (1.5639, 3.4023, 0, 134.35)	0.25
Apr.	5048	Gamma (29.437, 171.48)	95.01	Gamma (5.0585, 18.783)	0.5
May.	6875	Invgauss (6875, 403354.6)	186.79	Gamma (21.196, 8.8125)	0.75
Jun.	15129	Gamma (55.408, 273.04)	183.67	Gamma (23.587, 7.7869)	0.75
Jul.	28026	Invgauss (28026, 446227)	158.24	Gamma (2.0237, 78.193)	0.75
Aug.	22841	Gamma (15.983, 1429.1)	117.37	Pearson5 (18.948, 2106.5)	0.75
Sep	17362	Gamma (84.86, 204.59)	175.78	Lognormal (175.78, 77.922)	0.75
Oct.	13050	Gamma (444.79, 29.34)	61.97	Kumaraswamy (4.2422, 10.109, 0, 119.21)	0.5
Nov.	6601	Invgauss (6601.1, 1093626.8)	56.34	Invgauss (56.341, 674.625)	0.5
Dec.	5227	Weibull (9.5305, 5505.6)	13.54	Uniform (0, 27.073)	0.25

[a]The reservoir inflow discharge in Chongqing section of the TGR was monitored every month from the year 2009 to 2012 (9 sites).

[b]The data were based on China Meteorological Data Service Center (CMDC) (http://sthjj.cq.gov.cn/).

Table S4 Quantitative microbial risk assessment parameters and assumptions.

Parameter (unit)	Symbol	Mean value	Probability distribution	Reference
fraction of (oo)cyst removed by STP (%)	F_{rem}	66.81	BetaGeneral (1.063, 0.52806, 0, 100)	(Cheng et al. 2009; Lim et al. 2007; Madore et al. 1987; Medema and Schijve 2001; Nasse 2016; Neto et al. 2006; Ottoson et al. 2006; Robertson et al. 2000; Taran-Benshoshan et al. 2015)
Fraction of human feces applied as fertilizer after storage (%)	$F_{s,h}$	85.4	Weibull (25.291, 87.227)	(Liu et al. 2014)
Fraction of animal manure applied as fertilizer after storage (%)	$F_{s,a}$	78.17	Gamma (37.864, 2.0646)	(Qiu et al. 2012; Zeng et al. 2013)
Manure storage time (days)	t_s	152	Uniform (30, 274)	(Liu et al. 2019; Vermeulen et al. 2017)
Cattle prevalence *C.parvum* and *C. hominis* (%)	s_a	52	Uniform (49, 55)	(Khan et al. 2010; Limaheluw et al. 2019; Robertson et al. 2014)
Sheep and goats prevalence *C.parvum* and *C. hominis* (%)		35	Uniform (20, 51)	(Khan et al. 2010; Limaheluw et al. 2019; Robertson et al. 2014)
Cattle prevalence *G. lamblia* assemblage A or B (%)		55	Uniform (19, 90)	(Feng and Xiao 2011; Abeywardena et al. 2013, 2014)
Sheep and goats prevalence *G. lamblia* assemblage A or B (%)		5	Fixed value	(Feng and Xiao 2011)
Total sown area in Chongqing (hectare)	S_{area}	3515790	Fixed value	Chong Qing statistical yearbook 2014 (http://tjj.cq.gov.cn/tjsj/shuju/tjnj/)
Concentration of oocyst in the TGR (oocyst/10L)	$C_{s,\ m}$	6.34	Lognormal (6.34, 14.08)	In this study
Concentration of cyst in the TGR (cyst/10L)		0.81	Lognormal (0. 81, 1.839)	In this study
Reduction factor for transport in soil	f_{soil}	1.5	Unifom (1, 2)	(Atwill et al. 2002; Santamaría et al. 2011)
Depth of groundwater (m)	D_{ground}	85.5	Unifom (1, 170)	(Fan et al. 2008; Pi et al. 2002; Zhang et al. 2018b; Fujino et al. 2006)
Concentration of oocyst in the groundwater (oocyst/10L)	$C_{g,\ m}$	0.0035	Lognormal (0.0035, 0.017)	In this study
Concentration of cyst in the groundwater (cyst/10L)		0.00104	Lognormal (0.00104, 0.0034)	In this study
Drinking water treatment efficiency (log_{10})				
Conventional	R_c	2.15	Triangular (2.03, 2.15, 2.27)	(Han et al. 2020)
Ozone	R_o	2.42	Gamma (2.0352, 1.1909)	(Cummins et al. 2010)

Parameter (unit)	Symbol	Mean value	Probability distribution	Reference
Microfiltration	R_{mf}	2.9	Uniform (2.3,3.5)	(Xiao et al. 2012)
Fraction infectious oocysts (%)	FI	90	Beta (9, 1)	(Xiao et al. 2018)
Fraction infectious cysts (%)		100	Beta (4.99, 0.01)	(Xiao et al.. 2018)
Water ingestion volume	V_i			
Swimming in fresh water (L/time)		-	Lognorm distribution	See Table S5 for more detail
Drinking unboiled tap water for children (≤ 4 years) (L/day)		0.53	Lognorm (0.53026, 0.1073)	
Drinking unboiled tap water for children (5-9 years) (L/day)		0.82	Lognorm (0.8238, 0.14512)	
Drinking unboiled tap water for children (10-14 years) (L/day)		1.01	Lognorm (1.0124, 0.061638)	
Drinking unboiled tap water for adults (15-64 years) (L/day)		1.50	Lognorm (1.501, 0.11178)	
Drinking unboiled tap water for adults (≥ 65 years) (L/day)		1.28	Lognorm (1.2799, 0.079698)	
Incidental intake of unboiled tap water (L/day)		0.039	Fixed value	(An et al. 2011)
Infectivity constant of *Crypt.*	r	0.09	Lognorm (0.09, 0.01)	(USEPA 2006)
Infectivity constant of *Giar.*		0.059	Lognorm (0.059, 0.033)	(Han et al. 2020)
Sensitivity parameters to pathogens in different age groups	S_j	-	Fixed value	See Table S1 for more detail
Infection-enhancing coefficient of independent effect of *Crypt.* and *Giar.*	f_k	1	Fixed value	(Han et al. 2020)
Infection-enhancing coefficient of synergistic effect of HIV for *Crypt.*		4.235	Uniform (0.57, 7.9)	(Tellevik et al. 2015; Yang et al. 2017)
Infection-enhancing coefficient of synergistic effect of HIV for *Giar.*		2.41	Uniform (1.23, 3.59)	(Feitosa et al. 2001)
Frequency of exposures per year	T_{year}			
Swimming in freshwater (times)		96	Fixed value	(Han et al. 2020)
Drinking unboiled tap water (days)		365	Fixed value	(An et al. 2011)
Incidental intake of unboiled tap water (days)		365	Fixed value	(An et al. 2011)
Immunocompetent probability of illness given infection of *Crypt.*	$P_{ill,inf}$	0.71	Beta (20, 8)	(Xiao et al. 2012)

Parameter (unit)	Symbol	Mean value	Probability distribution	Reference
Immunocompetent probability of illness given infection of *Giar.*		0.45	Uniform (0.2, 0.7)	(Chhipi-Shrestha et al. 2017)
Immunodeficiency probability of illness given infection		1	Fixed value	(Xiao et al. 2012)
Immunocompetent case fatality ratio for *Crypt.*	P_{cfr}	0.00001	Beta (1, 99999)	(Havelaar and Mels 2003)
Immunocompetent case fatality ratio for *Giar.*		0	Fixed value	(Gibney et al. 2014)
Immunodeficiency case fatality ratio		0.03	Beta (3, 97)	(Dietz et al. 2000)
Fraction of exposure population	F			
Swimmers (%)		-	Lognorm distribution	See Table S5 for more detail
Populations who drinking unboiled tap water (%)		4.45	Fixed value	(Peng et al. 2008)
Populations who ingesting unboiled tap water by incidental intake (%)		100	Fixed value	(An et al. 2011)
Proportions of acute cryptosporidiosis	P_{sym}			
Mild		0.86	Lognorm(0.86, 0.001)	(Gibney et al. 2014)
Outpatient		0.123	Lognorm(0.123, 0.001)	(Gibney et al. 2014)
Hospitalized		0.017	Lognorm(0.017, 0.001)	(Gibney et al. 2014)
Proportions of acute giardiasis				
Mild		0.905	Lognorm(0.905, 0.001)	(Gibney et al. 2014)
Outpatient		0.093	Lognorm(0.093, 0.001)	(Gibney et al. 2014)
Hospitalized		0.002	Lognorm(0.002, 0.001)	(Gibney et al. 2014)
Duration of acute cryptosporidiosis (day)	L			
Mild		4	Lognorm(4, 2)	(Gibney et al. 2014)
Outpatient		12.5	Lognorm(12.5, 3)	(Gibney et al. 2014)
Hospitalized		21.4	Lognorm(21.4, 7)	(Gibney et al. 2014)
Duration of acute giardiasis (day)				
Mild		5	Lognorm(5, 3)	(Gibney et al. 2014)
Outpatient		15	Lognorm(15, 4)	(Gibney et al. 2014)
Hospitalized		33	Lognorm(33, 10)	(Gibney et al. 2014)
Disability weights of acute gastroenteritis	W			
Mild		0.061	Lognorm(0.061, 0.0188)	(Salomon et al. 2012)
Outpatient		0.202	Lognorm(0.202, 0.0405)	(Salomon et al. 2012)
Hospitalized		0.281	Lognorm(0.281, 0.0792)	(Salomon et al. 2012)

Table S5 Age and swimming habits of Chinese people.

Age	Proportion of swimmers (Wang and Duan 2016; Zhao and Duan 2013,2016)		Volume of swimming ingestion (L/min) (Dufour et al. 2006)		Swimming duration (min/visits) (Wang and Duan 2016; Zhao and Duan 2013,2016)	
	Mean (95% CI)	Distributions	Mean (95% CI)	Distributions	Mean (95% CI)	Distributions
≤4	0.062652 (0.03590-0.09935)	Lognorm (0.062652, 0.01986)	0.000822	Fixed value	8.7169 (5.963-12.158)	Lognorm (8.7169, 1.9099)
5-9	0.14844 (0.07861-0.24809)	Lognorm (0.14844, 0.053479)	0.000822	Fixed value	19.218 (13.031-26.988)	Lognorm (19.218, 4.3061)
10-14	0.19479 (0.16685-0.22551)	Lognorm (0.19479, 0.017877)	0.000822	Fixed value	27.202 (27.105-27.299)	Lognorm (27.202, 0.058814)
15-64	0.045955 (0.01308-0.10725)	Lognorm (0.045955, 0.032672)	0.0003818	Lognorm (0.0003818, 0.0000766302)	20.61 (16.656-25.109)	Lognorm (20.61, 2.5818)
≥ 65	0.0059087 (0.001866-0.013152)	Lognorm (0.0059087, 0.0038401)	0.000356	Fixed value	17.334 (12.656-22.974)	Lognorm (17.334, 3.1676)

[a] Water ingestion volume of swimming (L/time) use formula volume of swimming ingestion ＊ swimming duration.

Table S6 Monthly mean concentrations of (oo)cysts in surface water (TGR) in Chongqing.

Month	*Cryptosporidium* (oocysts/10L)			*Giardia* (cysts/10L)		
	Mean ± SD	Min. - Max.	95% CI	Mean ± SD	Min. - Max.	95% CI
Jan.	13.3 ± 8.3	1.0-73.1	4.1-29.9	3.5 ± 2.3	0.2-24.2	0.9-7.9
Feb.	19.3 ±11.7	1.4-88.7	6.2-42.8	5.0 ±3.3	0.3-32.7	1.4-11.5
Mar.	16.4 ± 10.9	1.0-138.8	4.8-37.6	4.1 ±3.0	0.2-30.6	1.0-10.1
Apr.	8.4 ± 6.3	0.0-53.5	1.2-20.5	2.4 ±2.0	0.0-22.4	0.3-6.2
May.	2.4 ± 2.0	0.1-31.1	0.6-6.2	0.7 ± 0.7	0.0-8.3	0.1-2.0
Jun.	1.1 ± 0.9	0.0-10.1	0.1-2.8	0.3 ± 0.3	0.0-3.8	0.0-0.9
Jul.	0.7 ± 0.6	0.0-5.8	0.1-1.8	0.2 ± 0.2	0.0-2.7	0.0-0.5
Aug.	0.5 ± 0.6	0.0-7.7	0.1-1.6	0.2 ± 0.2	0.0-2.4	0.0-0.5
Sep	0.6 ± 0.6	0.0-5.5	0.0 -1.7	0.2 ± 0.2	0.0-2.3	0.0-0.6
Oct.	1.9 ± 1.4	0.1-14.5	0.5-4.5	0.5 ± 0.4	0.0-4.3	0.1-1.4
Nov.	4.8 ± 3.9	0.0-48.1	0.9-12.2	1.4 ± 1.2	0.0-14.2	0.2-3.8
Dec.	10.1 ± 6.6	0.7-53.7	2.9-23.3	2.6 ± 1.9	0.1-17.8	0.7-6.3

[a] Values were based on the results from 10,000 model iterations.

Table S7 Monthly mean concentrations of (oo)cysts in groundwater in Chongqing.

Month	*Cryptosporidium* (oocysts/10L)			*Giardia* (cysts/10L)		
	Mean ± SD	Min. - Max.	95% CI	Mean ± SD	Min. - Max.	95% CI
Jan.	0.28±0.25	0-361.10	$0\text{-}2.64\times10^{-11}$	0.093±0.14	0-106.84	$0\text{-}3.41\times10^{-11}$
Feb.	0.15±0.16	0-257.76	$0\text{-}1.9\times10^{-11}$	0.058±0.08	0-108.92	$0\text{-}1.56\times10^{-11}$
Mar.	0.083±0.17	0-265.89	$0\text{-}1.19\times10^{-11}$	0.045±0.042	0-60.93	$0\text{-}2.06\times10^{-12}$
Apr.	0.027±0.012	0-96.58	$0\text{-}3.03\times10^{-12}$	0.005±0.007	0-8.19	$0\text{-}1.16\times10^{-12}$
May.	0.008±0.005	0-15.41	$0\text{-}1.08\times10^{-12}$	0.002±0.003	0-2.69	$0\text{-}8.4\times10^{-13}$
Jun.	0.007±0.004	0-11.11	$0\text{-}1.13\times10^{-12}$	0.007±0.002	0-48.73	$0\text{-}4.24\times10^{-13}$
Jul.	0.007±0.015	0-9.46	$0\text{-}1.81\times10^{-12}$	0.004±0.006	0-6.48	$0\text{-}2.20\times10^{-13}$
Aug.	0.007±0.005	0-20.41	$0\text{-}1.49\times10^{-12}$	0.005±0.004	0-14.10	$0\text{-}6.83\times10^{-13}$
Sep	0.008±0.006	0-6.77	$0\text{-}6.12\times10^{-13}$	0.003±0.003	0-3.85	$0\text{-}1.05\times10^{-12}$
Oct.	0.023±0.017	0-34.17	$0\text{-}6.82\times10^{-12}$	0.012±0.010	0-11.56	$0\text{-}1.29\times10^{-12}$
Nov.	0.028±0.028	0-46.68	$0\text{-}6.5\times10^{-12}$	0.014±0.014	0-24.86	$0\text{-}2.83\times10^{-12}$
Dec.	0.16±0.62	0-157.11	$0\text{-}2.59\times10^{-11}$	0.19±0.017	0-502.83	$0\text{-}1.13\times10^{-11}$

[a] Values were based on the results from 10,000 model iterations.

Table S8 Manure excretion by humans and different livestock species in China.

Parameter (kg day^{-1})	Symbol	Mean value	Probability distribution	Reference
Human	M_h	0.40	Uniform (0.31, 0.50)	(Liu et al. 2019; Huang et al. 2012)
Cattle	$M_{a,\ cattle}$	21.88	Gamma (163.39, 0.13)	(Peng et al. 2016; Liu et al. 2018; Huang et al. 2017; Zhu et al. 2014; Bao et al. 2018; Wang et al. 2018; Wang et al. 2006; Liang et al. 2013; Chen et al. 2009; Chadwick et al. 2015; Liu et al. 2019)
Sheep and goats	$M_{a,\ sheep\ \ and\ goats}$	2.03	Weibull (6.70, 2.18)	(Liu et al. 2018; Huang et al. 2017; Bao et al. 2018; Wang et al. 2018; Wang et al. 2006; Liang et al. 2013; Chadwick et al. 2015; Chen et al. 2009; Liu et al. 2019; Guo et al. 2011)
Pigs	$M_{a,\ pigs}$	3.24	Weibull (7.84, 3.44)	(Peng et al. 2016; Huang et al. 2017; Zhu et al. 2014; Bao et al. 2018; Wang et al. 2018; Liang et al. 2013; Chadwick et al. 2015; Chen et al. 2009; Liu et al. 2019)
Laying hens	$M_{a,\ l\text{aying hens}}$	0.13	Gamma (30.78, 0.004)	(Peng et al. 2016; Liu et al. 2018; Huang et al. 2017; Zhu et al. 2014; Wang et al. 2018; Wang et al. 2006; Liang et al. 2013; Chadwick et al. 2015; Chen et al. 2009; Liu et al. 2019)
Broilers	$M_{a,b\text{roilers}}$	0.09	Weibull (6.17, 0.10)	(Peng et al. 2016; Liu et al. 2018; Huang et al. 2017; Zhu et al. 2014; Wang et al. 2018; Wang et al. 2006; Liang et al. 2013; Chadwick et al. 2015; Chen et al. 2009; Liu et al. 2019)
Ducks and goose	$M_{a,\ d\text{ucks and goose}}$	0.13	Weibull (15.70, 0.13)	(Wang et al. 2006; Zhang et al. 2012; Bao et al. 2018; Liang et al. 2013; Chadwick et al. 2015; Chen et al. 2009; Liu et al. 2019)
Rabbits	$M_{a,\ r\text{abbits}}$	0.24	Uniform (0.11,0.37)	(Zhu et al. 2014; Bao et al. 2018; Wang et al. 2006; Guo et al. 2011)

Table S9 Model input for *Cryptosporidium* oocyst and *Giardia* cyst excretion rates for humans and livestock.

Pathogens	Parameter	Symbol	Mean value	Probability distribution	Reference
Cryptosporidium (oocysts g^{-1})	Human	$O_{h,\ Cryp}$	9073	Uniform (7638, 10509)	(Chappell et al. 1999; Liu et al. 2019)
	Cattle	$O_{a,\ \mathrm{cattle},\ Cryp}$	241	Gamma(0.42, 574.38)	(Atwill et al. 2006; Graczyk et al. 2000; Hutchison et al. 2005; Yun and Lee 2000; Ferguson et al. 2007; Daniels et al. 2015)
	Sheep and goats	$O_{a,\ \mathrm{sheep\ and\ goats},\ Cryp}$	276	Gamma (0.46, 606.57)	(Cox et al. 2005; Hutchison et al. 2005; Vermeulen et al. 2017; Ferguson et al. 2007; Daniels et al. 2015)
	Pigs	$O_{a,\ \mathrm{pigs},\ Cryp}$	176	Gamma (1.55, 113.89)	(Hutchison et al. 2005; Cox et al. 2005; Ferguson et al. 2007; Vermeulen et al. 2017; Dorner et al. 2004)
	Laying hens	$O_{a,\ \mathrm{laying\ hens},\ Cryp}$	8604	Gamma (0.80, 10826)	(Hornok et al. 2000, 1998a, 1998b; Ferguson et al. 2007; Vermeulen et al. 2017; Rhee et al. 1998; Sréter et al. 1995; Hornok et al. 1999; Varga et al. 1995; Rhee et al. 1995; Dorner et al 2004)
	Broilers	$O_{a,\mathrm{broilers},\ Cryp}$	8604	Gamma (0.80, 10826)	(Hornok et al. 1998; Hornok et al. 1998b; Hornok et al. 2000; Rhee et al. 1998; Sréter et al. 1995; Hornok et al. 1999; Varga et al. 1995; Rhee et al. 1995; Dorner et al. 2004; Ferguson et al. 2007; Vermeulen et al. 2017)
	Ducks and goose	$O_{a,\ \mathrm{ducks\ and\ goose},\ Cryp}$	18601	Uniform (50, 37153)	(Ferguson et al. 2007; Vermeulen et al. 2017; Rhee et al. 1995; Dorner et al. 2004)
	Rabbits	$O_{a,\ \mathrm{rabbits},\ Cryp}$	6	Fixed value	(Ferguson et al. 2007)
Giardia (cysts g^{-1})	Human	$O_{h,\ Giar}$	1018	Fixed value	(Chappell et al. 1999; Liu et al. 2019; Daniels et al. 2015)
	Cattle	$O_{a,\ \mathrm{cattle},\ Giar}$	507	Uniform (169, 844)	(Ferguson et al. 2007; Daniels et al. 2015)
	Sheep and goats	$O_{a,\ \mathrm{sheep\ and\ goats},\ Giar}$	459	Uniform (436, 482)	(Ferguson et al. 2007; Daniels et al. 2015)
	Pigs	$O_{a,\ \mathrm{pigs},\ Giar}$	524	Fixed value	(Ferguson et al. 2007)
	Laying hens	$O_{a,\ \mathrm{laying\ hens},\ Giar}$	436	Fixed value	(Ferguson et al. 2007)
	Broilers	$O_{a,\mathrm{broilers},\ Giar}$	436	Fixed value	(Ferguson et al. 2007)
	Ducks and goose	$O_{a,\ \mathrm{ducks\ and\ goose},\ Giar}$	436	Fixed value	(Kuhn et al. 2002)
	Rabbits	$O_{a,\ \mathrm{rabbits},\ Giar}$	0	Fixed value	(Ferguson et al. 2007)

Table S10 *Cryptosporidium* and *Giardia* prevalence in the feces of humans and livestock in China.

Pathogens	Parameter (%)	Symbol	Mean value	Probability distribution	Reference
Cryptosporidium	Human	$P_{h,\ Cryp}$	3.44	BetaGeneral (3.30, 9.05, 0, 13.00)	(Wang et al. 2015; Zhou et al. 2005 ; Zhang et al. 2012)
	Cattle	$P_{a,\ \mathrm{cattle},\ Cryp}$	14.94	BetaGeneral (2.19, 13.58, 0, 100)	(Cai et al. 2019; Liu et al. 2009; Ma et al. 2015; Wang et al. 2011; Yang et al. 2012; Zhang et al. 2015)
	Sheep and goats	$P_{a,\ \mathrm{sheep\ and\ goats},\ Cryp}$	6.79	BetaGeneral (6.42, 87.82, 0, 100)	(Mi et al. 2014; Wang et al. 2014; Zhu et al. 2018; Li et al. 2019; Li et al. 2019)
	Pigs	$P_{a,\ \mathrm{pigs},\ Cryp}$	20.48	BetaGeneral (2.79, 11.53, 0, 100)	(Chen et al. 2011; Wang et al. 2010b; Yang et al. 2012; Yin et al. 2011; Zou et al. 2017; Yin et al. 2013)
	Laying hens	$P_{a,\ \mathrm{laying\ hens},\ Cryp}$	6.52	Uniform (2.43, 10.60)	(Cao et al. 2020; Liao et al. 2018; Wang et al. 2010a)
	Broilers	$P_{a,\mathrm{broilers},\ Cryp}$	4.72	Uniform (2.43, 7.00)	(Liao et al. 2018; Wang et al. 2010a; Cao et al. 2020)
	Ducks and goose	$P_{a,\ \mathrm{ducks\ and\ goose},\ Cryp}$	10.20	Uniform (4.10, 16.30)	(Zhao et al. 2019; Wang et al. 2010a)
	Rabbits	$P_{a,\ \mathrm{rabbits},\ Cryp}$	2.5	Uniform (1.60, 3.40)	(Shi et al. 2010; Zhang et al. 2013; Zhang et al. 2018; Zhang et al. 2012)
Giardia	Human	$P_{h,\ Giar}$	6.20	BetaGeneral (1.9951, 30.166, 0, 100)	(Liu et al. 2014; Wang et al. 2017; Ma et al. 1994; Sun et al. 2012)
	Cattle	$P_{a,\ \mathrm{cattle},\ Giar}$	7.47	BetaGeneral (1.2169, 15.076, 0, 100)	(Cui et al. 2018; Jian et al. 2018; Li et al. 2016; Wang et al. 2014, 2019; Zhang et al. 2016)
	Sheep and goats	$P_{a,\ \mathrm{sheep\ and\ goats},\ Giar}$	9.38	BetaGeneral (1.0701, 10.34, 0, 100)	(Chang et al. 2019; Chen et al. 2019; Zhang et al. 2012; Zhong et al. 2018)
	Pigs	$P_{a,\ \mathrm{pigs},\ Giar}$	7.72	BetaGeneral (0.68148, 8.1451, 0, 100)	(Jing et al. 2019; Liu et al. 2019; Wang et al. 2018; Zhang et al. 2019; Zou et al. 2019)
	Laying hens	$P_{a,\ \mathrm{laying\ hens},\ Giar}$	8.25	Fixed value	(Cao et al. 2020)
	Broilers	$P_{a,\mathrm{broilers},\ Giar}$	8.25	Fixed value	(Cao et al. 2020)
	Ducks and goose	$P_{a,\ \mathrm{ducks\ and\ goose},\ Giar}$	28	Fixed value	(Kuhn et al. 2002)

Table S11 The breeding days for different livestock species in China.

Parameter (days)	Symbol	Mean value	Probability distribution	Reference
Cattle	D_{cattle}	365	Fixed value	(Liu et al. 2019; Li et al. 2009; Shi et al. 2014; Wang et al. 2018; Zhao et al. 2016; Jing et al. 2012; Li et al. 2019) (http://sthjj.cq.gov.cn/)
Sheep and goats	$D_{sheep\ and\ goats}$	345	Gamma (47.847, 7.2035)	(Liu et al. 2019; Li et al. 2009; Shi et al. 2014; Wang et al. 2018; Zhao et al. 2016) (http://sthjj.cq.gov.cn/)
Sows and gilts for breeding	$D_{sows\ and\ gilts\ for\ breeding}$	365	Fixed value	(Liu et al. 2019; Wang et al. 2018; Jing et al. 2012)(http://sthjj.cq.gov.cn/)
Boars	D_{boars}	200	Gamma (34.185, 5.8473)	(Liu et al. 2019; Li et al. 2009; Zhou et al. 2010; Shi et al. 2014; Wang et al. 2018; Zhao et al. 2016; Jing et al. 2012; Li et al. 2019) (http://sthjj.cq.gov.cn/)
Laying hens	$D_{laying\ hens}$	339	Gamma (26.867, 12.624)	(Liu et al. 2019; Shi et al. 2014; Wang et al. 2018; Zhao et al. 2016; Li et al. 2019) (http://sthjj.cq.gov.cn/)
Broilers	$D_{broilers}$	59	Gamma (26.954, 2.1843)	(Liu et al. 2019; Zhou et al. 2010; Shi et al. 2014; Wang et al. 2018; Zhao et al. 2016; Jing et al. 2012; Li et al. 2019) (http://sthjj.cq.gov.cn/)
Ducks and goose	$D_{ducks\ and\ goose}$	60	Uniform (50, 70)	(Zhao et al. 2016; Jing et al. 2012)
Rabbits	$D_{rabbits}$	98	Uniform (55, 140)	(Liu et al. 2019; Zhou et al. 2010; Zhao et al. 2016) (http://sthjj.cq.gov.cn/)

Table S12 The statistics of monthly air temperature during the year 2013 in Chongqing section of the TGR.

Month (T_m)	Mean value (℃) [a]	Probability distribution
Jan.	4.88	BetaGeneral (9.4215, 25.396, -10, 45)
Feb.	8.06	BetaGeneral (13.896, 28.43, -10, 45)
Mar.	14.15	BetaGeneral (11.539, 14.736, -10, 45)
Apr.	16.64	BetaGeneral (12.936, 13.768, -10, 45)
May.	20.06	BetaGeneral (19.612, 16.272, -10, 45)
Jun.	24.43	BetaGeneral (18.38, 10.979, -10, 45)
Jul.	27.17	BetaGeneral (20.923, 10.038, -10, 45)
Aug.	26.73	BetaGeneral (17.389, 8.65, -10, 45)
Sep	20.48	BetaGeneral (25.999, 20.893, -10, 45)
Oct.	16.73	BetaGeneral (19.024, 20.126, -10, 45)
Nov.	11.51	BetaGeneral (20.153, 31.378, -10, 45)
Dec.	5.90	BetaGeneral (10.784, 26.527, -10, 45)

[a]The data were based on China Meteorological Data Service Center (CMDC) (http://sthjj.cq.gov.cn/).

Table S13 The statistics of monthly water temperature and water level during the year 2009-2012 in Chongqing section of the TGR.

Month	Water temperature ($T_{w,m}$)[a]		Water level (Z_m) [a]	
	Mean value (°C)	Probability distribution	Mean value (m)	Probability distribution
Jan.	13.4	Logistic (13.35404, 0.34122)	172.8	BetaGeneral (13.064, 0.88367, 140, 175)
Feb.	11.5	Expon (0. 5)	168.5	BetaGeneral (69.788, 15.795, 140, 175)
Mar.	13.1	Uniform (11.5065, 14.5935)	160.4	BetaGeneral (10.44, 7.4647, 140, 175)
Apr.	17.3	Gamma (2.3384, 1.5404)	157.1	BetaGeneral (9.048, 9.4448, 140, 175)
May.	22.0	ExtvalueMin (22.5814, 1.0105)	155.6	BetaGeneral (18.068, 22.347, 140, 175)
Jun.	24.2	Loglogistic (21.2815, 2.5596, 3.5307)	152.3	BetaGeneral (2.2615, 4.1504, 140, 175)
Jul.	25.4	Triang (22.8601, 24, 29.3986)	152.2	BetaGeneral (2.8705, 5.3945, 140, 175)
Aug.	26.3	Normal (26.3185, 1.7097)	149.1	BetaGeneral (23.224, 66.236, 140, 175)
Sep	26.3	Triang (23.3, 23.3, 32.1906)	155.8	BetaGeneral (2.2174, 2.6864, 140, 175)
Oct.	21.8	Normal (21.8, 1.2765)	168.6	BetaGeneral (6.4012, 1.4331, 140, 175)
Nov.	19.1	Logistic (19.0603, 1.0573)	173.4	BetaGeneral (11.31, 0.53788, 140, 175)
Dec.	15.3	Logistic (15.30474, 0.72022)	173.6	BetaGeneral (10.869, 0.45279, 140, 175)

[a]The water temperature and water level in Chongqing section of the TGR was monitored every month from the year 2009 to 2012 (9 sites).

Table S14 The statistics of monthly surface solar radiation and dissolved organic carbon during the year 2013 in Chongqing section of the TGR.

Month	Surface solar radiation ($I_{A,m}$) [a]		Dissolved organic carbon ($C_{DOC,m}$) [b]	
	Mean value ($KJ^{-2}day^{-1}$)	Probability distribution	Mean value (mg L^{-1})	Probability distribution
Jan.	5239	Uniform (0, 10478)	0.64	Fixed value
Feb.	5767	Gamma (1.7047, 3382.9)	1.82	Fixed value
Mar.	11056	Weibull (2.4031, 12471)	2.12	Fixed value
Apr.	14051	Weibull (2.0809, 15863)	1.33	Fixed value
May.	12653	Gamma (1.4653, 8635.4)	6.67	Fixed value
Jun.	18638	Kumaraswamy (0.7721, 0.40566, 0, 27920)	4.11	Fixed value
Jul.	19124	BetaGeneral (1.9951, 0.71936, 0, 26020)	5.19	Fixed value
Aug.	17948	Kumaraswamy (1.3216, 0.41107, 0, 23810)	5.16	Fixed value
Sep	13750	BetaGeneral (1.0579, 0.60776, 0, 21650)	4.70	Fixed value
Oct.	13325	BetaGeneral (2.2864, 0.99774, 0, 19140)	3.32	Fixed value
Nov.	3311	Invgauss (3310.7, 3907.6)	1.12	Fixed value
Dec.	916	Weibull (4.58, 1003.1)	1.50	Fixed value

[a]The data were based on China Meteorological Data Service Center (CMDC) (http://sthjj.cq.gov.cn/), as there is not enough data in September, October and December in Chongqing, we used the same values from Wuhan and Hubei province.

[b]The data were based on literature (Huang et al. 2017).

Fig. S1 Distribution of disease burden in immunocompetent (outer ring) and immunodeficient subpopulations (inner ring) attributable to infection by *Cryptosporidium* and *Giardia* via consumption of surface and ground waters in Chongqing, by severity category.

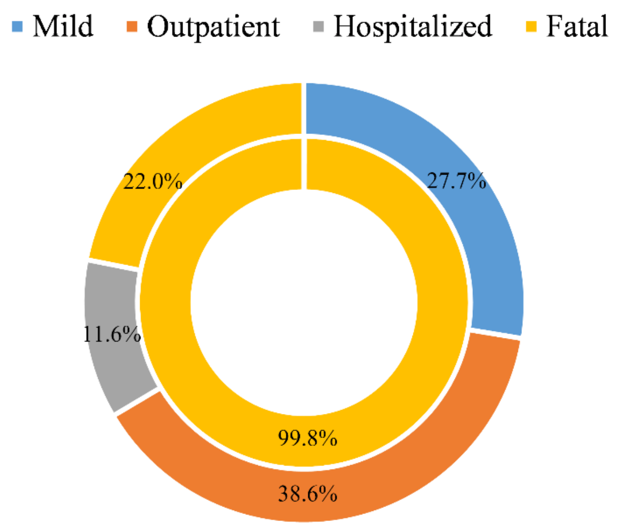

Fig. S2 Tornado chart showing Spearman rank order correlation coefficients between the GloWPa-TGR-Crypt-Giar C1 model parameters and the *Cryptosporidium* (A) and *Giardia* (B) concentrations in Chongqing section of the TGR caused by the human and livestock feces discharge.

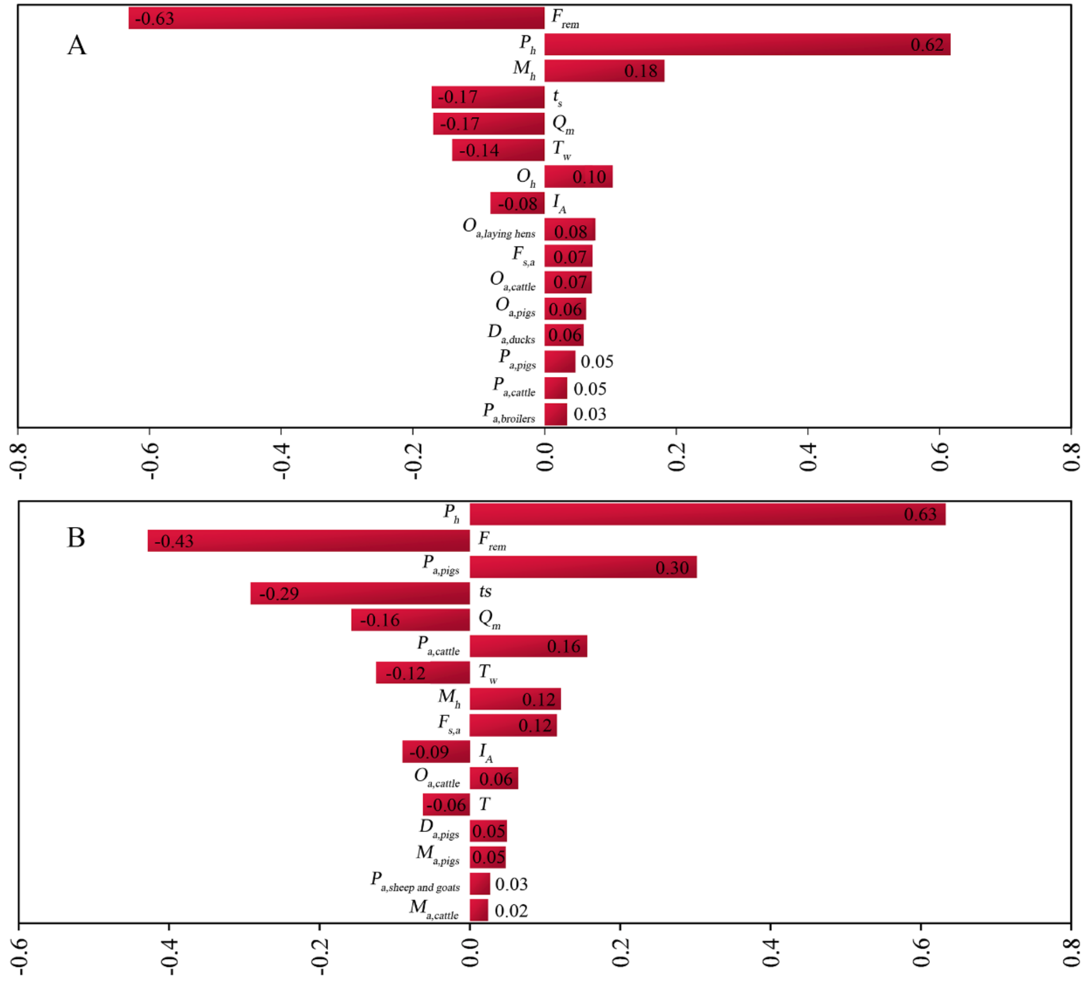

Fig. S3 Tornado chart showing Spearman rank order correlation coefficients between the GloWPa-TGR-Crypto C1 model parameters and the *Cryptosporidium* (A) and *Giardia* (B) concentrations in tubewells of Chongqing caused by the human and livetock feces discharge.

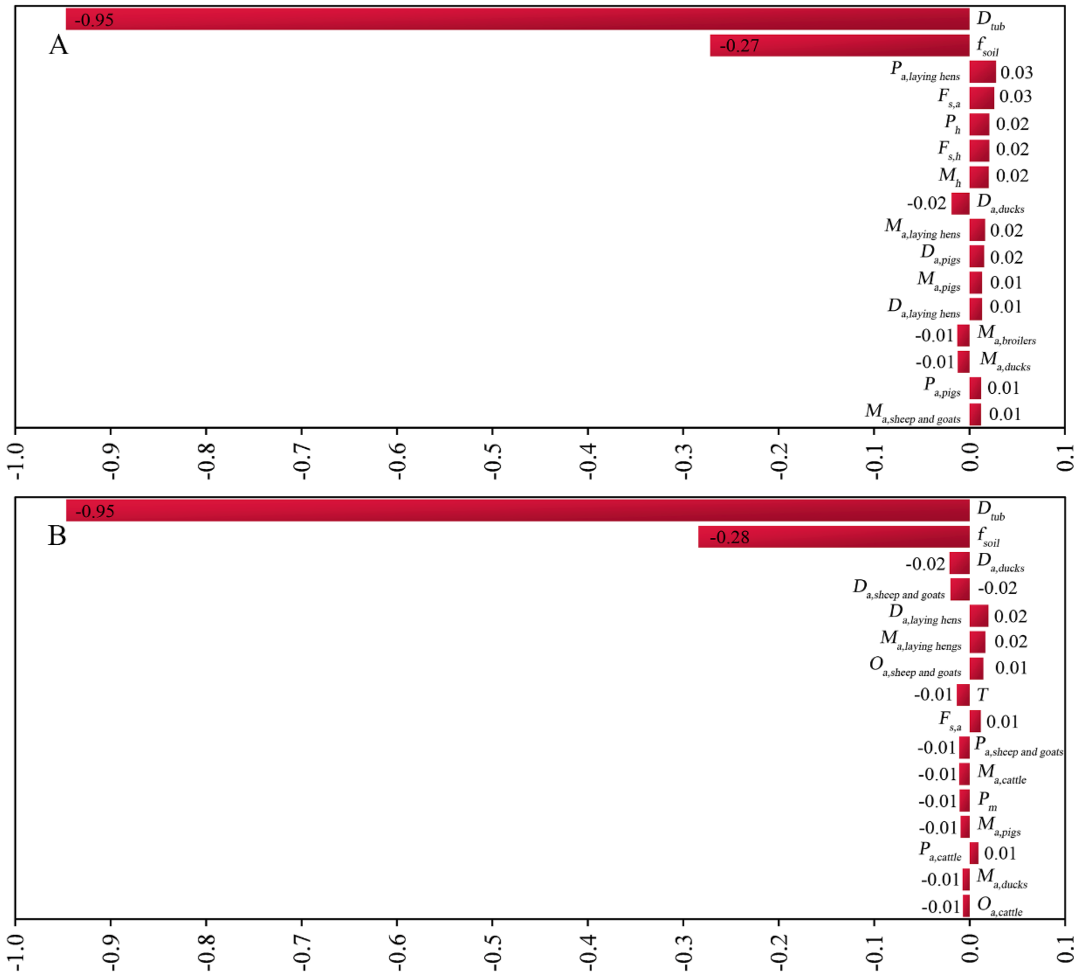

Fig.S4 Tornado chart showing Spearman rank order correlation coefficients between the QMRA model parameters and the burden of disease in DALYs attributable to *Cryptosporidium* and *Giardia* in consumed the surface and ground water in the TGR watershed of Chongqing (conventional treatment).

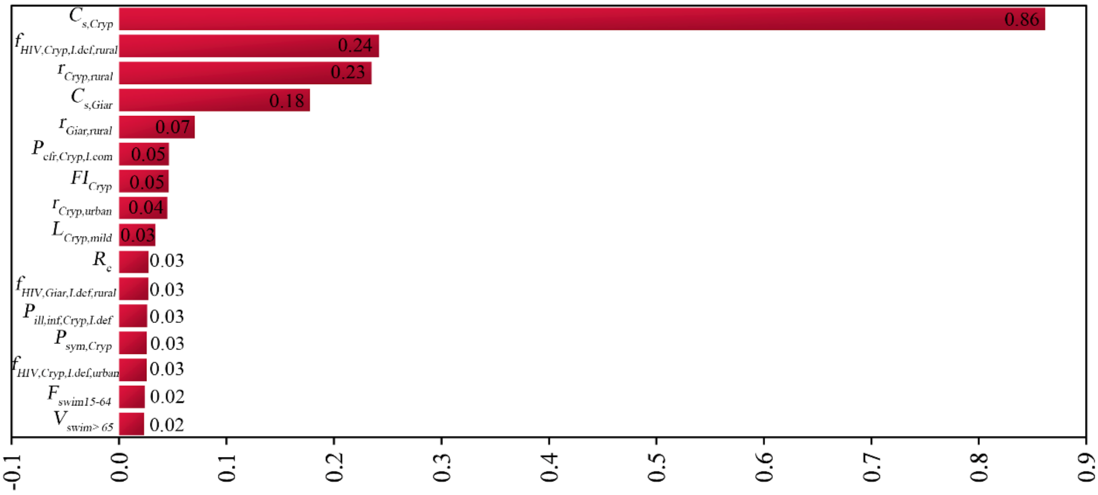

Reference

Havelaar AH, Havelaar JMMelse (2003) Quantifying Public Health Risk in the WHO Guidelines for Drinking Water Quality. WHO RIVM Rep. 1–49.

Abeywardena H, Jex AR, Koehler A V et al (2014) First molecular characterization of Cryptosporidium and Giardia from bovines (Bos taurus and Bubalus bubalis) in Sri Lanka: Unexpected absence of C. parvum from pre-weaned calves. Parasites and Vectors 7: 1–10. https://doi.org/10.1186/1756-3305-7-75

Abeywardena H, Jex AR, von Samson-Himmelstjerna G et al (2013) First molecular characterisation of Cryptosporidium and Giardia from Bubalus bubalis (water buffalo) in Victoria, Australia. Infect Genet Evol 20: 96–102. https://doi.org/10.1016/j.meegid.2013.07.019

An W, Zhang D, Xiao S et al (2011) Quantitative health risk assessment of cryptosporidium in rivers of Southern China based on continuous monitoring. Environ Sci Technol 45: 4951–4958. https://doi.org/10.1021/es103981w

Atwill ER, Hou L, Karle BM et al (2002) Transport of Cryptosporidium parvum oocysts through vegetated buffer strips and estimated filtration efficiency. Appl Environ Microbiol 68: 5517–5527. https://doi.org/10.1128/AEM.68.11.5517-5527.2002

Atwill ER, Pereira MDGC, Alonso LH et al (2006) Environmental Load of Cryptosporidium parvum Oocysts from Cattle Manure in Feedlots from the Central and Western United States . J Environ Qual 35: 200–206. https://doi.org/10.2134/jeq2005.0099

Bao W, Liu J, An J et al (2018) Discussion on value-taking of relative parameters for assessment of livestock and poultry excrement resource in China. Transactions of the Chinese Society of Agricultural Engineering. 34: 314-322 (in Chinese). https://doi.org/10.11975/j.issn.1002-6819.2018.24.038

Cai Y, Zhang NZ, Gong QL et al (2019) Prevalence of Cryptosporidium in dairy cattle in China during 2008–2018: A systematic review and meta-analysis. Microb Pathog 132: 193–200. https://doi.org/10.1016/j.micpath.2019.05.006

Cao S, Xu M, Jiang Y et al (2020) Prevalence and Genetic Characterization of Cryptosporidium, Giardia and Enterocytozoon in Chickens From Ezhou, Hubei, China. Front Vet Sci 7: 1–7. https://doi.org/10.3389/fvets.2020.00030

Chadwick D, Wei J, Yan’an T et al (2015) Improving manure nutrient management towards sustainable agricultural intensification in China. Agric Ecosyst Environ 209: 34–46. https://doi.org/10.1016/j.agee.2015.03.025

Chang Y, Wang Y, Wu Y et al (2019) Molecular Characterization of Giardia duodenalis and Enterocytozoon bieneusi Isolated from Tibetan Sheep and Tibetan Goats Under Natural Grazing Conditions in Tibet. J Eukaryot Microbiol 67:100-106 . https://doi.org/10.1111/jeu.12758

Chappell CL, Okhuysen PC, Sterling CR et al (1999) Infectivity of Cryptosporidium parvum in healthy adults with pre- existing anti-C. parvum serum immunoglobulin G. Am J Trop Med Hyg 60: 157–164. https://doi.org/10.4269/ajtmh.1999.60.157

Chen W, Liu D, Liu J (2009) Study on livestock carrying capacity based on manure nutrients. Chinese Journal of Animal Science 45: 46-50 (in Chinese).

Chen Y, Li X, Liu X et al (2019) Tele-connecting China’s future urban growth to impacts on ecosystem services under the shared socioeconomic pathways. Sci Total Environ 652: 765–779. https://doi.org/10.1016/j.scitotenv.2018.10.283

Chen Z, Mi R, Yu H et al (2011) Prevalence of Cryptosporidium spp. in pigs in Shanghai, China. Vet Parasitol 181: 113–119. https://doi.org/10.1016/j.vetpar.2011.04.037

Cheng HWA, Lucy FE, Graczyk TK et al (2009) Fate of Cryptosporidium parvum and Cryptosporidium hominis oocysts and Giardia duodenalis cysts during secondary wastewater treatments. Parasitol Res 105: 689–696. https://doi.org/10.1007/s00436-009-1440-y

Cheng L, Deng H, He S (2019) Distribution patterns and diversity of plant communities in fluctuating areas of Chongqing section of Yangtze River. Chinese Journal of Ecology 38: 3626-3634 (in Chinese). https://doi.org/10.13292/j.1000-4890.201912.033

Chhipi-Shrestha G, Hewage K, Sadiq R (2017) Microbial quality of reclaimed water for urban reuses: Probabilistic risk-based investigation and recommendations. Sci Total Environ 576: 738–751. https://doi.org/10.1016/j.scitotenv.2016.10.105

Cox P, Griffith M, Angles M et al (2005) Concentrations of pathogens and indicators in animal feces in the Sydney watershed. Appl Environ Microbiol 71: 5929–5934. https://doi.org/10.1128/AEM.71.10.5929-5934.2005

Cui P, Ma X, Li H et al (2018) Shared biological pathways between Alzheimer's disease and ischemic stroke. Front Neurosci 12: 605. https://doi.org/10.3389/fnins.2018.00605

Cummins E, Kennedy R, Cormican M (2010) Quantitative risk assessment of Cryptosporidium in tap water in Ireland. Sci Total Environ 408: 740–753. https://doi.org/10.1016/j.scitotenv.2009.11.008

Daniels ME, Shrivastava A, Smith WA et al (2015) Cryptosporidium and giardia in humans, domestic animals, and village water sources in rural India. Am J Trop Med Hyg 93: 596–600. https://doi.org/10.4269/ajtmh.15-0111

Dietz V, Vugia D, Nelson R et al (2000) Active, multisite, laboratory based surveillance for Cryptosporidium parvum. American Journal of Tropical Medicine and Hygiene 62: 368e372. https://doi.org/10.4269/ajtmh.2000.62.368

Dorner SM, Huck PM, Slawson RM (2004) Estimating potential environmental loadings of Cryptosporidium spp. and Campylobacter spp. from livestock in the Grand River Watershed, Ontario, Canada. Environ Sci Technol 38: 3370–3380. https://doi.org/10.1021/es035208+

Dufour AP, Evans O, Behymer TD et al (2006) Water ingestion during swimming activities in a pool: a pilot study. J Water Health 4: 425e430.

Fan H, Su C, Wang Y et al (2008) Sedimentary arsenite-oxidizing and arsenate-reducing bacteria associated with high arsenic groundwater from Shanyin, Northwestern China. J Appl Microbiol 105: 529–539. https://doi.org/10.1111/j.1365-2672.2008.03790.x

Feitosa G, Bandeira AC, Sampaio DP et al (2001) High prevalence of giardiasis and stronglyloidiasis among HIV-infected patients in Bahia, Brazil. Braz J Infect Dis 5: 339–344. https://doi.org/10.1590/s1413-86702001000600008

Ferguson CM, Croke BF, Beatson PJ et al (2007) Development of a process-based model to predict pathogen budgets for the Sydney drinking water catchment. J Water Health 5:187-208. https://doi.org/10.2166/wh.2007.013

Fujino Y, Guo X, Shirane K et al (2006) Arsenic in drinking water and peripheral nerve conduction velocity among residents of a chronically arsenic-affected area in Inner Mongolia. J Epidemiol 16: 207-13. https://doi.org/10.2188/jea.16.207

Fu M, Sun Q, Sun L (2004) Investigation on Giardia bamblia infection among students in Huainan. Chinese Journal of School Doctor 31: 474-478 (in Chinese). https://doi.org/10.3969/j.issn.1001-7062.2004.02.041

Gibney KB, O'Toole J, Sinclair M et al (2014) Disease burden of selected gastrointestinal pathogens in Australia, 2010. Int J Infect Dis 28: 176–185. https://doi.org/10.1016/j.ijid.2014.08.006

Guo D, Wu H, Ma Y et al (2011) Study on the amount of manure and urine excreted by sheep and rabbits in intensive pasture. Journal of Ecology & Rural Environment 27: 44-48 (in Chinese). https://doi.org/10.1016/S1671-2927(11)60313-1

Graczyk TK, Evans BM, Shiff CJ et al (2000) Environmental and geographical factors contributing to watershed contamination with Cryptosporidium parvum oocysts. Environ Res 82: 263–271. https://doi.org/10.1006/enrs.1999.4022

Han M, Xiao S, An W et al (2020) Co-infection risk assessment of Giardia and Cryptosporidium with HIV considering synergistic effects and age sensitivity using disability-adjusted life years. Water Res 175: 115698. https://doi.org/10.1016/j.watres.2020.115698

Hornok S, Bitay Z, Széll Z et al (1998a) Assessment of maternal immunity to Cryptosporidium baileyi in chickens. Vet Parasitol 79: 203–212. https://doi.org/10.1016/S0304-4017(98)00170-8

Hornok S, Heijmans JF, Békési L et al (1998b) Interaction of chicken anaemia virus and Cryptosporidium baileyi in experimentally infected chickens. Vet Parasitol 76: 43–55. https://doi.org/10.1016/S0304-4017(97)00046-0

Hornok S, Széll Z, Shibalova TA et al (1999) Study on the course of cryptosporidium baileyi infection in chickens treated with interleukin-1 or indomethacin. Acta Vet Hung 47: 207–216. https://doi.org/10.1556/004.47.1999.2.6

Hornok S, Széll Z, Sréter T et al (2000) Influence of in ovo administered Cryptosporidium baileyi oocyst extract on the course of homologous infection. Vet Parasitol 89: 313–319. https://doi.org/10.1016/S0304-4017(00)00217-X

Huang J, Chen L (2012) Benefit evaluation of biogas production from human and livestock manure in rural areas of Yunnan Province. Southwest China Journal of Agricultural Sciences 25: 1884-1888 (in Chinese). https://doi.org/10.3969/j.issn.1001-4829.2012.05.070

Huang M, Xia Y, Fan X (2017) Evaluation of livestock pollution status in the Three Gorges Reservoir Area of Hubei Province. Chinese Journal of Ecology 36: 725-733 (in Chinese). https://doi.org/10.13292/j.1000-4890.201703.019

Huang Q, Yang L, Li B et al (2020) Cryptosporidium spp. And Giardia duodenalis emissions from humans and animals in the Three Gorges Reservoir in Chongqing, China. PeerJ 8: 1–27. https://doi.org/10.7717/peerj.9985

Hutchison ML, Walters LD, Avery SM et al (2005) Analyses of livestock production, waste storage, and pathogen levels and prevalences in farm manures. Appl Environ Microbiol 71: 1231–1236. https://doi.org/10.1128/AEM.71.3.1231-1236.2005

Jian Y, Zhang X, Li X et al (2018) Prevalence and molecular characterization of Giardia duodenalis in cattle and sheep from the Qinghai-Tibetan Plateau Area (QTPA), northwestern China. Vet Parasitol 250: 40–44. https://doi.org/10.1016/j.vetpar.2017.12.001

Jing B, Zhang Y, Xu C et al (2019) Detection and genetic characterization of Giardia duodenalis in pigs from large-scale farms in Xinjiang, China. Parasite 26: 53. https://doi.org/10.1051/parasite/2019056

Jing D, Chen X, Yu H (2012) Analysis on the total amount of domestic animal excrement and the load in farmland in Foshan. Journal of Ecology and Rural Environment 28: 108-111(in Chinese). https://doi.org/10.3969/j.issn.1673-4831.2012.01.019

Khan SM, Debnath C, Pramanik AK et al (2010) Molecular characterization and assessment of zoonotic transmission of Cryptosporidium from dairy cattle in West Bengal, India. Vet Parasitol 171: 41–47. https://doi.org/10.1016/j.vetpar.2010.03.008

Kuhn RC, Rock CM, Oshima KH (2002) Occurrence of Cryptosporidium and Giardia in wild ducks along the Rio Grande River valley in Southern New Mexico. Appl Environ Microbiol 68: 161–165. https://doi.org/10.1128/AEM.68.1.161-165.2002

Li F, Wang H, Zhang Z et al (2016) Prevalence and molecular characterization of Cryptosporidium spp. and Giardia duodenalis in dairy cattle in Beijing, China. Vet Parasitol 219: 61–65. https://doi.org/10.1016/j.vetpar.2016.01.023

Li FY, Wu X, Li J et al (2019) Assessment of carbon sequestration and nutrient resources by turning animal manure into biochar and its potential environmental risk for field application. J Agro-Environment Sci 38: 2202–2209. https://doi.org/10.11654/jaes.2019-0025

Li P, Li Y, Yang D (2009) Estimation of annual discharge of livestock and poultry manure in Tianjin. Animal husbandry and veterinary medicine 41: 32-34 (in Chinese).

Li P, Tu R, Cao J et al (2019) Analysis of Cryptosporidium infection and genotype identification in goat under different breeding modes. Animal husbandry and veterinary medicine 51: 91-94 (in Chinese).

Li WC, Wang K, Tang L et al (2019) Molecular characterization of Cryptosporidium species in sheep and goats in Anhui Province and neighboring provinces. Zhongguo Xue Xi Chong Bing Fang Zhi Za Zhi 31: 474-478 (in Chinese). https://doi.org/10.16250/j.32.1374.2018043

Liang L, Lal R, Du Z et al (2013) Estimation of nitrous oxide and methane emission from livestock of urban agriculture in Beijing. Agric Ecosyst Environ 170: 28–35. https://doi.org/10.1016/j.agee.2013.02.005

Liao C, Wang T, Koehler AV et al (2018) Molecular investigation of Cryptosporidium in farmed chickens in Hubei Province, China, identifies “zoonotic” subtypes of C. meleagridis. Parasites and Vectors 11: 1–8. https://doi.org/10.1186/s13071-018-3056-5

Lim YA, Wan Hafiz WI, Nissapatorn V (2007) Reduction of Cryptosporidium and Giardia by sewage treatment processes. Trop Biomed 24: 95–104. https://doi.org/10.1016/j.pt.2007.04.005

Limaheluw J, Medema G, Hofstra N (2019) An exploration of the disease burden due to Cryptosporidium in consumed surface water for sub-Saharan Africa. Int J Hyg Environ Health 222: 856–863. https://doi.org/10.1016/j.ijheh.2019.04.004

Liu A, Wang R, Li Y et al (2009) Prevalence and distribution of Cryptosporidium spp. in dairy cattle in Heilongjiang Province, China. Parasitol Res 105: 797–802. https://doi.org/10.1007/s00436-009-1457-2

Liu C (2018) Estimation environmental impact of livestock and poultry manure in Hunan. Heilongjiang Animal Science and Veterinary Medicine 5: 72-74 (in Chinese). https://doi.org/10.13881/j.cnki.hljxmsy.2017.07.0345

Liu W, An W, Jeppesen E et al (2019) Modelling the fate and transport of Cryptosporidium, a zoonotic and waterborne pathogen, in the Daning River watershed of the Three Gorges Reservoir Region, China. J Environ Manage 232: 462–474. https://doi.org/10.1016/j.jenvman.2018.10.064

Liu Y, Huang JK, Zikhali P (2014) Use of Human Excreta as Manure in Rural China. J Integr Agric https://doi.org/10.1016/S2095-3119(13)60407-4

Liu Y, Huang JK, Zikhali P (2014) Use of human excreta as manure in rural China. J Integr Agric 13: 434–442.

Ma C, Zhang Q, Fu D (1994) Human parasitic infections in children and juveniles in Ningxia. Chinese Journal of Parasitology& Parasitic Diseases. (S1): 92-94 (in Chinese).

Ma J, Li P, Zhao X et al (2015) Occurrence and molecular characterization of Cryptosporidium spp.

and Enterocytozoon bieneusi in dairy cattle, beef cattle and water buffaloes in China. Vet Parasitol 207: 220–227. https://doi.org/10.1016/j.vetpar.2014.10.011

Madore MS, Rose JB, Gerba CP et al (1987) Occurrence of Cryptosporidium oocysts in sewage effluents and selected surface waters. J Parasitol 73: 702–705. https://doi.org/10.2307/3282398

Medema GJ, Schijven JF (2001) Modelling the sewage discharge and dispersion of cryptosporidium and giardia in surface water. Water Res 35: 4307–4316. https://doi.org/10.1016/S0043-1354(01)00161-0

Mi R, Wang X, Huang Y et al (2014) Prevalence and molecular characterization of Cryptosporidium in goats across four provincial level areas in China. PLoS One 9: 1–7. https://doi.org/10.1371/journal.pone.0111164

Nasser AM (2016) Removal of Cryptosporidium by wastewater treatment processes: A review. J Water Health 14: 1–13. https://doi.org/10.2166/wh.2015.131

Neto RC, Santos JU, Franco RMB (2006) Evaluation of activated sludge treatment and the efficiency of the disinfection of Giardia species cysts and cryptosporidium oocysts by UV at a sludge treatment plant in Campinas, south-east Brazil. Water Sci Technol 54: 89–94. https://doi.org/10.2166/wst.2006.453

Office E (2008) Summary of the joint assessment report on China’s AIDS prevention and control (2007). Chin Nurs Manag 8: 9-11.

Ottoson J, Hansen A, Westrell T et al (2006) Removal of noro- and enteroviruses, Giardia cysts, Cryptosporidium oocysts, and fecal indicators at four secondary wastewater treatment plants in Sweden. Water Environ Res 78: 828–834. https://doi.org/10.2175/106143006x101719

Peng J, Yang L, Bao Z (2016) The estimation of production amount of animal manure and its energy and environmental effect in Tianjin. Acta Ecologae Animalis Domastici 37: 65-68 (in Chinese). https://doi.org/10.3969/j.issn.1673-1182.2016.10.013

Peng X, Murphy T, Holden NM (2008) Evaluation of the effect of temperature on the die-off rate for Cryptosporidium parvum oocysts in water, soils, and feces. Appl Environ Microbiol 74: 7101–7107. https://doi.org/10.1128/AEM.01442-08

Pi J, Yamauchi H, Kumagai Y et al (2002) Evidence for induction of oxidative stress caused by chronic exposure of Chinese residents to arsenic contained in drinking water. Environ. Health Perspect 110: 331–336. https://doi.org/10.1289/ehp.02110331

Qiu H, Mo H, Bai J (2012) Treatment and influencing factors of livestock and poultry manure in rural China: An empirical analysis based on survey data from five provinces. Chinese Rural Economy. 3: 78-87 (in Chinese).

Rhee JK, Jang BG, Park BK (1995) Oocyst production and immunogenicity of Cryptosporidium baileyi in chickens and mallards. Korean J Parasitol 33: 45-54. https://doi.org/10.3347/kjp.1995.33.1.45

Rhee JK, Kim HC, Park BK (1998) Effect of Cryptosporidium baileyi infection on antibody response to sRBC in chickens. Korean J Parasitol 36: 33-6. https://doi.org/10.3347/kjp.1998.36.1.33

Robertson LJ, Björkman C, Axén C et al (2014) Cryptosporidiosis in Farmed Animals. In: Cacciò, S., Widmer, G. (eds) Cryptosporidium: parasite and disease. Springer, Vienna. https://doi.org/10.1007/978-3-7091-1562-6_4

Robertson LJ, Paton CA, Campbell AT et al (2000) Giardia cysts and Cryptosporidium oocysts at sewage treatment works in Scotland, UK. Water Res 34: 2310–2322. https://doi.org/10.1016/S0043-1354(99)00408-X

Salomon JA, Vos T, Hogan DR et al (2012) Common values in assessing health outcomes from disease and injury: Disability weights measurement study for the Global Burden of Disease Study 2010. Lancet 380: 2129–2143. https://doi.org/10.1016/S0140-6736(12)61680-8

Shi J, Zhou S, Zhao J et al (2014) Livestock and poultry manure excrements and their environmental effect evaluation in Nanyang. Acta Ecologae Animalis Domastici 35: 76-81(in Chinese). https://doi.org/10.3969/j.issn.1673-1182.2014.12.015

Shi K, Jian F, Lv C et al (2010) Prevalence, genetic characteristics, and zoonotic potential of Cryptosporidium species causing infections in farm rabbits in China. J Clin Microbiol 48: 3263–3266. https://doi.org/10.1128/JCM.00819-10

Sréter T, Varga I, Békési L (1995) Age-dependent resistance to Cryptosporidium baileyi infection in chickens. J Parasitol 81: 827-9.

Sun X, Wang J, liang G (2012) Risk factors of Giardia Lamblia infection in residents at both sides of China-Myanmar border area of Nabang port,Yunnan province. Chinese Journal of Public Health 28: 320-322.

Sun Y, Zhang F, Hu H et al (2014) Statistical analysis of influent quality characteristics of municipal wastewater treatment plants in Chongqing. Chinese Journal of Environmental Engineering 8: 5167-5173 (in Chinese).

Taran-Benshoshan M, Ofer N, Dalit VO et al (2015) Cryptosporidium and Giardia removal by secondary and tertiary wastewater treatment. J Environ Sci Heal 50: 1265–1273. https://doi.org/10.1080/10934529.2015.1055152

Tellevik MG, Moyo SJ, Blomberg B (2015) Prevalence of Cryptosporidium parvum/hominis, Entamoeba histolytica and Giardia lamblia among Young Children with and without Diarrhea in Dar es Salaam, Tanzania. PLoS Negl Trop Dis 9: 1–16. https://doi.org/10.1371/journal.pntd.0004125

USEPA (2006) National Primary Drinking Water Regulations: Long Term 2 Enhanced Surface Water Treatment Rule (LT2ESWTR); Final Rule.

Varga I, Sréter T, Békési L (1995) Quantitative method to assess Cryptosporidium oocyst shedding in the chicken model. Parasitol Res 81: 262–264. https://doi.org/10.1007/BF00937120

Velthof GL, Oudendag D, Witzke HP et al (2009) Integrated assessment of nitrogen losses from agriculture in EU-27 using MITERRA-EUROPE. J Environ Qual 38: 402–417. https://doi.org/10.2134/jeq2008.0108

Vermeulen LC, Benders J, Medema G et al (2017) Global Cryptosporidium Loads from Livestock Manure. Environ Sci Technol 51: 8663–8671. https://doi.org/10.1021/acs.est.7b00452

Vermeulen LC, van Hengel M, Kroeze C et al (2019) Cryptosporidium concentrations in rivers worldwide. Water Res 149: 202–214. https://doi.org/10.1016/j.watres.2018.10.069

Wang BB, Duan XL (2016) Exposure Factors Handbook of Chinese Population (Children 0-5 Years). China Environmental Press, BeiJing.

Wang H, Zhang Y, Wu Y et al (2018) Occurrence, Molecular Characterization, and Assessment of Zoonotic Risk of Cryptosporidium spp., Giardia duodenalis, and Enterocytozoon bieneusi in Pigs in Henan, Central China. J Eukaryot Microbiol 65: 893–901. https://doi.org/10.1111/jeu.12634

Wang F, Ma W, Dou Z et al (2006) The estimation of the production amount of animal manure and its environmental effect in China. China Environmental Science 26: 614-617 (in Chinese). https://doi.org/10.3321/j.issn:1000-6923.2006.05.024

Wang M, Zhang S, Yang J et al (2018) Livestock manure emission and cultivated land pollution load in

the midst upstream of Danjiang River in Shangluo City. Chinese Journal of Eco-Agriculture 26:1898-1907 (in Chinese). https://doi.org/10.13930/j.cnki.cjea.180174

Wang QQ, Guo JD, Cao ZG et al (2015) Investigation on human Cryptosporidium infection in local area of Anhui Province. Chinese J Schistosomiasis Control 27: 1–6. https://doi.org/10.16250/j.32.1374.2015049

Wang R, Jian F, Sun Y et al (2010a) Large-scale survey of Cryptosporidium spp. in chickens and Pekin ducks (Anas platyrhynchos) in Henan, China: Prevalence and molecular characterization. Avian Pathol 39: 447–451. https://doi.org/10.1080/03079457.2010.518314

Wang R, Li G, Cui B et al (2014) Prevalence, molecular characterization and zoonotic potential of Cryptosporidium spp. in goats in Henan and Chongqing, China. Exp Parasitol 142: 11–16. https://doi.org/10.1016/j.exppara.2014.04.001

Wang R, Ma G, Zhao J et al (2011) Cryptosporidium andersoni is the predominant species in post-weaned and adult dairy cattle in China. Parasitol Int 60: 1–4. https://doi.org/10.1016/j.parint.2010.09.002

Wang R, Qiu S, Jian F et al (2010b) Prevalence and molecular identification of Cryptosporidium spp. in pigs in Henan, China. Parasitol Res 107: 1489–1494. https://doi.org/10.1007/s00436-010-2024-6

Wang SS, Yuan YJ, Yin YL et al (2017) Prevalence and multilocus genotyping of Giardia duodenalis in pigs of Shaanxi Province, northwestern China. Parasites and Vectors 10: 1–8. https://doi.org/10.1186/s13071-017-2418-8

Wang Y, Yin D, Xiang Y et al (2019) A Review of Studies on the Biogeochemical Behaviors of Mercury in the Three Gorges Reservoir, China. Bull Environ Contam Toxicol 102: 686–694. https://doi.org/10.1007/s00128-019-02586-1

Xiao S, An W, Chen Z et al (2012) The burden of drinking water-associated cryptosporidiosis in China: The large contribution of the immunodeficient population identified by quantitative microbial risk assessment. Water Res 46: 4272–4280. https://doi.org/10.1016/j.watres.2012.05.012

Xiao S, Yin P, Zhang Y et al (2018) Occurrence, genotyping, and health risk of Cryptosporidium and Giardia in recreational lakes in Tianjin, China. Water Res 141: 46–56. https://doi.org/10.1016/j.watres.2018.05.016

Yang Y, Chen Y, Zhang X et al (2012) Methodology for agricultural and rural NPS pollution in a typical county of the North China Plain. Environ Pollut 168: 170–176. https://doi.org/10.1016/j.envpol.2012.04.017

Yang Y, Zhou YB, Xiao PL et al (2017) Prevalence of and risk factors associated with Cryptosporidium infection in an underdeveloped rural community of Southwest China. Infect Dis Poverty 6: 1–10. https://doi.org/10.1186/s40249-016-0223-9

Yaoyu F, Xiao L (2011) Zoonotic potential and molecular epidemiology of Giardia species and giardiasis. Clin Microbiol Rev 24: 110–140. https://doi.org/10.1128/CMR.00033-10

Yin J, Shen Y, Yuan Z et al (2011) Prevalence of the Cryptosporidium pig genotype II in pigs from the Yangtze River Delta, China. PLoS One 6: 1–4. https://doi.org/10.1371/journal.pone.0020738

Yin JH, Yuan ZY, Cai HX et al (2013) Age-related infection with Cryptosporidium species and genotype in pigs in china. Biomedical & Environmental Sciences 26: 492-495. https://doi.org/10.3967/0895-3988.2013.06.010

Yun EJ, Lee YN (2000) Estimating maximum possible environmental loading amounts of

Cryptosporidium parvum attributable to adult beef cattle. Quant Microbiol 2: 21–36. https://doi.org/10.1023/A:1010044012356

Zeng J, Shu J, Che Y et al (2013) The pollution management survey and countermeasures of rural livestock and poultry industry in Chongqing. Journal of Guizhou University (Natural Science) 30: 126-129 (in Chinese). https://doi.org/10.3969/j.issn.1000-5269.2013.05.028

Zhang HJ, Song JK, Wu XM et al (2019) First report of Giardia duodenalis genotypes in Zangxiang pigs from China. Parasitol Res 118: 2305–2310. https://doi.org/10.1007/s00436-019-06340-8

Zhang R, Li X, Li H et al (2012) Cryptosporidium in eastern Chongqing. Parasitoses and Infectious Diseases 10: 72-74 (in Chinese).

Zhang T, Bu M, Geng W (2012) Pollution status and biogas-producing potential of livestock and poultry excrements in China. Chinese Journal of Ecology 31: 1241-1249 (in Chinese).

Zhang W, Yang F, Liu A et al (2013) Prevalence and Genetic Characterizations of Cryptosporidium spp. in Pre-Weaned and Post-Weaned Piglets in Heilongjiang Province, China. PLoS One 8: e67564. https://doi.org/10.1371/journal.pone.0067564

Zhang W, Zhang XL, Wang R et al (2012) Genetic Characterizations of Giardia duodenalis in Sheep and Goats in Heilongjiang Province, China and Possibility of Zoonotic Transmission. PLoS Negl Trop Dis 6:e1826. https://doi.org/10.1371/journal.pntd.0001826

Zhang X, Qi M, Jing B et al (2018) Molecular Characterization of Cryptosporidium spp., Giardia duodenalis, and Enterocytozoon bieneusi in Rabbits in Xinjiang, China. J Eukaryot Microbiol 65: 854–859. https://doi.org/10.1111/jeu.12629

Zhang XX, Tan QD, Zhao GH et al (2016) Prevalence, Risk Factors and Multilocus Genotyping of Giardia intestinalis in Dairy Cattle, Northwest China. J Eukaryot Microbiol 63: 498–504. https://doi.org/10.1111/jeu.12293

Zhang Y, Chen Z, An W et al (2015) Risk assessment of Giardia from a full scale MBR sewage treatment plant caused by membrane integrity failure. J Environ Sci (China) 30: 252–258. https://doi.org/10.1016/j.jes.2014.09.033

Zhang Y, Wu Y, Sun J et al (2018) Controls on the spatial distribution of iodine in groundwater in the Hebei Plain, China. Environ Sci Pollut Res 25: 16702–16709. https://doi.org/10.1007/s11356-018-1843-3

Zhao J, Chen H, Yang H et al (2019) Science of the Total Environment Epigenetic silencing of ALX4 regulates microcystin-LR induced hepatocellular carcinoma through the P53 pathway. Sci Total Environ 683: 317–330. https://doi.org/10.1016/j.scitotenv.2019.05.144

Zhao J, Yin C (2016) Analysis on the total amount of domestic animal excrement and the potential of fertilizer utilization in Qingdao city. Chinese Journal of Agricultural Resources and Regional Planning 37: 108-115 (in Chinese). https://doi.org/10.7621/cjarrp.1005-9121.20160716

Zhao XG, Duan XL (2013) Exposure Factors Handbook of Chinese Population (Adults). China Environmental Press, BeiJing.

Zhao XG, Duan XL (2016) Exposure Factors Handbook of Chinese Population (Children 6-17 Years). China Environmental Press, BeiJing.

Zhong Z, Tu R, Ou H et al (2018) Occurrence and genetic characterization of Giardia duodenalis and Cryptosporidium spp. from adult goats in Sichuan Province, China. PLoS One 13: 1–11. https://doi.org/10.1371/journal.pone.0199325

Zhou H, Zhu M, Yuan J et al (2005) A survey on Cryptosporidium parvum cryptosporidiosis in different people of Luwan district Shanghai. Shanghai Journal of Preventive Medicine 17: 430-

432 (in Chinese). https://doi.org/10.3969/j.issn.1004-9231.2005.09.012
Zhou K, Lei Z, Wan Z et al (2010) Estimation of annual total livestock/poultry excrement in Henan Province. Chinese Journal of Eco-Agriculture 18: 1060-1065 (in Chinese). https://doi.org/10.3724/SP.J.1011.2010.01060
Zhu J, Zhang Z, Fan Z et al (2014) Biogas potential, crop land load and total amount control of animal manure in China. Journal of Agro-Environment Science 33: 435-445 (in Chinese). https://doi.org/10.11654/jaes.2014.03.005
Zhu W, Mi R, Wang J et al (2018) Detection and actin sequence analysis of Cryptosporidium infection in sheep and goats in Tengzhou, Shandong Province. Chinese Journal of Veterinary Parasitology 26: 69-75 (in Chinese).
Zou Y, Ma JG, Yue DM et al (2017) Prevalence and risk factors of Cryptosporidium infection in farmed pigs in Zhejiang, Guangdong, and Yunnan provinces, China. Trop Anim Health Prod 49: 653–657. https://doi.org/10.1007/s11250-017-1230-y
Zou Y, Zheng W, Song HY et al (2019) Prevalence and genetic characterization of Enterocytozoon bieneusi and Giardia duodenalis in Tibetan pigs in Tibet, China. Infect Genet Evol 75: 104019. https://doi.org/10.1016/j.meegid.2019.104019
